# Thermomechanically Coupled Modeling and Process-Parameter Analysis of Laser-Field-Assisted Hybrid Milling of GH4096 Superalloy

**DOI:** 10.3390/mi17101166

**Published:** 2026-10-07

**Authors:** Hongyang Yan, Hongqin Lei, Ping Liang, Haodi Zhao, Yunlei Wang, Zhenyu Wang, Jian Cheng, Henan Liu, Shicheng Zhou, Linjie Zhao, Mingjun Chen

**Affiliations:** 1 State Key Laboratory of Robotics and System, Harbin Institute of Technology, Harbin 150001, China; 2 Zhengzhou Advanced Research Institute, Harbin Institute of Technology, Zhengzhou 450000, China; 3 Beijing Power Machinery Institute, Beijing 100074, China

**Keywords:** laser-assisted milling, GH4096 superalloy, thermomechanical coupling, Johnson–Cook plasticity and damage, surface von mises equivalent stress, process-parameter analysis

## Abstract

GH4096 is a Chinese nickel-based disk superalloy used for disks and shafts in advanced gas-turbine engines, where creep, fatigue, corrosion resistance, and microstructural stability are service-critical. These requirements are associated with high deformation resistance and thermomechanical loading during milling. However, laser-assisted milling (LAM) of GH4096 has not yet been systematically investigated. This study addresses that material–process gap through a three-dimensional thermomechanically coupled finite-element analysis based on a GH4096 constitutive-damage description. Johnson–Cook (JC) plasticity and damage parameters are calibrated using quasi-static, high-strain-rate, elevated-temperature, and stress-state-dependent data, while displacement-based damage evolution is used after damage initiation. A moving Gaussian surface heat source reproduces localized preheating and coupled material removal. The model quantifies the effects of laser power, spot radius, cutting depth, feed speed, and spindle speed on milling force, the transient temperature field and surface von Mises equivalent stress. Intermittent force peaks occur in all three directions, with the largest local peaks arising mainly from axial *RF3* loading. Feed speed produces the largest peak-force variation relative to the baseline case (73.0%), followed by cutting depth (65.6%). Laser power, spot radius, and spindle speed produce variations of 29.7%, 28.8%, and 28.2%, respectively. Force peaks respond non-monotonically to laser power, spot radius, and spindle speed. Maximum surface von Mises equivalent stress also varies non-monotonically with all five parameters. Feed speed produces the largest fluctuation, peaking at 623.56 MPa at 20 mm/s before decreasing to 529.32 MPa at 30 mm/s. Cutting depth yields a minimum of 502.88 MPa at 0.5 mm and a maximum of 580.90 MPa at 0.9 mm. Surface von Mises equivalent stress peaks at 619.75 MPa at 400 W and decreases to 550.89 MPa at a 2.2 mm spot radius. It increases sharply at 2.4–2.6 mm and reaches 573.14 MPa at 10,000 r/min before declining. These results establish a bounded basis for coordinating optical and mechanical parameters in GH4096 LAM within the investigated model and parameter ranges.

## 1. Introduction

Nickel-based disk superalloys are indispensable in highly loaded aero-engine rotor assemblies, where disks and shafts must sustain high centrifugal stress, cyclic thermomechanical loading, oxidation and long dwell periods without losing dimensional stability or damage tolerance [1,2,3]. GH4096 is a Chinese nickel-based disk superalloy used for disks and shafts in advanced gas-turbine engines [2,3,4]. Its creep, fatigue and corrosion resistance, together with microstructural stability near 700 °C, are application-critical properties [2,3]. As in closely related FGH96-type disc alloys, the load-bearing microstructure consists of a γ matrix strengthened by coherent γ′ precipitates: coarse primary γ′ particles help stabilize grain boundaries, while finer secondary and tertiary precipitates impede dislocation motion; solution treatment and recrystallisation regulate their size, distribution and interaction with the matrix [5,6]. Prior deformation has also been shown to affect creep behavior in this related disc-alloy class, underscoring the importance of preserving the load-bearing substrate [7]. The same multiscale strengthening architecture that enables service performance makes GH4096 difficult to machine. High resistance to plastic flow, low heat dissipation from the cutting zone and pronounced near-surface work hardening expose an intermittently engaged milling edge to repeated mechanical and thermal shocks. Abrasion, adhesion and temperature-assisted diffusion can consequently promote coating loss, edge rounding and local chipping, while severe subsurface deformation may compromise the functional layer intended to remain in service [8,9]. A suitable process must therefore lower the deformation resistance of the material scheduled for removal while restricting thermal and mechanical damage to the load-bearing substrate.

In conventional milling, cutting velocity (*v_c_*), feed speed (*v_f_*) and depth of cut (*a_p_*) are balanced against removal efficiency, cutting load, temperature, tool durability and surface integrity. Alternative processes alter this balance in different ways [10]. Electrical discharge machining and electrochemical machining avoid direct mechanical shearing, but the former can create a recast and thermally affected layer, whereas the latter depends on controlled dissolution, electrolyte management and confinement of stray current [11,12]. Abrasive waterjet machining avoids a thermally transformed layer, although jet divergence, kerf taper and abrasive–surface interaction constrain dimensional and surface quality [13]. Where mechanical cutting must be retained, minimum-quantity lubrication modifies interfacial friction and heat transfer, cryogenic media intensify cooling, and ultrasonic assistance periodically changes tool–workpiece contact [14,15,16]. These routes are valuable for their intended functions, yet they do not impose a controlled traveling thermal field immediately ahead of each milling engagement. Laser-assisted milling (LAM) provides this capability by concentrating non-contact energy in the material about to enter the shear zone, allowing the heated volume and its residence time to be coordinated with tool motion while avoiding unnecessary bulk heating. Work on Inconel 718 associates beam–tool positioning with chip formation, cutting load, tool wear and machined-surface formation, and thermal-field modeling for GH4169 identifies laser power, beam radius, translation speed and beam–edge distance as coupled controls of local preheating [17,18]. To the best of our knowledge, no previous study has reported the application of LAM to GH4096. This material–process gap motivates a GH4096-specific analysis that preserves productive milling kinematics while introducing a spatially and temporally controllable thermal field before material removal.

Numerical analysis of this process requires a material description that remains tractable under large strain, high strain rate, rapid heating and repeated three-dimensional tooth engagement. The Zerilli–Armstrong model represents crystal-structure-dependent thermal and rate effects through dislocation mechanics, the mechanical threshold stress model evolves an internal resistance to thermally activated deformation, and the Preston–Tonks–Wallace model spans thermally activated and drag-controlled flow under extreme loading [19,20,21]. These formulations resolve different physical mechanisms and loading regimes, but their use for GH4096 would require material-specific parameterization; the evolving internal resistance in the mechanical threshold stress model adds a further calibration and integration demand. Johnson–Cook (JC) plasticity and JC damage initiation are established tools rather than innovations in themselves. For GH4096 laser-assisted milling, JC plasticity offers a practical balance: its compact multiplicative form separates strain hardening, logarithmic strain-rate sensitivity and homologous-temperature softening, its small parameter set can be identified through established mechanical-test routes, and its direct implementation in explicit finite-element solvers is efficient for severe deformation and frequent contact changes [22,23]. JC damage initiation complements this flow law by defining failure strain through stress triaxiality, strain rate and temperature [24]. In cutting simulation, the combined use of JC plasticity and damage supplies a practical mechanism for crack development and the creation of chip free surfaces [25]. In the present implementation, JC initiation is followed by a separately specified displacement-based damage evolution law that governs stiffness degradation before element deletion represents material separation. Modified JC formulations have also described recrystallisation-related softening in high-rate powder-metallurgy superalloys, demonstrating the extensibility of the JC formulation to this material class [26]. The relevant contribution in this study therefore does not lie in the individual JC or Gaussian heat-source formulation, but in their material-specific integration for simulating the coupled laser–thermal–mechanical-damage response of GH4096 during LAM. This distinction also limits the interpretation to the present coupled model and investigated parameter range.

This study makes two main contributions. First, this study establishes the engineering and material-specific basis for machining the GH4096 material investigated here, a Chinese nickel-based disk superalloy used for disks and shafts in advanced gas-turbine engines, by linking its service-critical property combination to a GH4096 constitutive–damage description calibrated from quasi-static, high-rate, elevated-temperature and stress-state-dependent data. Second, using three-dimensional coupled thermomechanical analysis, this study quantifies the effects of laser power, laser spot radius, cutting depth, feed speed, and spindle speed on milling force, the transient temperature field and surface von Mises equivalent stress, thereby filling a research gap in the application of laser-assisted milling to GH4096 superalloy.

## 2. Material Model Calibration and Finite Element Modeling of GH4096 Alloy for Laser-Assisted Milling

### 2.1. Johnson–Cook Plasticity Model and Parameter Identification

#### 2.1.1. Johnson–Cook Plasticity Model

Laser-assisted milling of GH4096 placed the material ahead of the cutting edge under rapid heating, large plastic shear and changing deformation rates during each engagement. The constitutive calculation therefore updated flow stress from the local strain, strain rate and temperature. This work used the Johnson–Cook (JC) plasticity relation to connect controlled GH4096 compression data with the material card implemented at each integration point. The adopted relation combined strain hardening, strain-rate strengthening, and thermal softening multiplicatively, as expressed in Equation (1) [23]:

(1) $$\sigma =(A+B\varepsilon^{n})(1+C\ln{\dot{\varepsilon }}^{*})(1-T^{*m})$$

where *σ* was the equivalent plastic flow stress. *ε* was the equivalent plastic strain. ${\dot{\varepsilon }}^{*}$ was the dimensionless equivalent plastic strain-rate ratio, which was defined by

(2) $${\dot{\varepsilon }}^{*}=\dot{\varepsilon }/{\dot{\varepsilon }}_{0}$$

The dimensionless temperature ratio T* was defined by

T^*^ = (*T* − *T*_0_)/(*T_m_* − *T*_0_) (3)

where *T*_0_ = 25 °C and *T*_m_ = 1280 °C. Temperatures were expressed on a consistent scale when evaluating T*. *A* defined the reference yield level; *B* and *n* defined strain hardening; *C* defined the logarithmic strain-rate term; and *m* defined thermal softening. Positive true compressive strain and positive equivalent stress were used throughout data processing and model input.

#### 2.1.2. Quasi-Static Compression Identification of A, B, and n

Quasi-static uniaxial compression was conducted at 25 °C with $\dot{\varepsilon }$_0_ = 0.001 s^−1^ as the target true strain rate. Cylindrical specimens with dimensions Ø 5 × 5 mm were machined from dense GH4096 ring material produced through hot isostatic pressing and isothermal forging. Precision wire electrical discharge machining controlled the specimen geometry, and both end faces were mechanically polished to set their parallel contact surfaces. A thin molybdenum disulfide layer was applied between the specimen and tungsten-carbide platens to control end friction during compression. These contact conditions and preparation were kept common for the curves used in the hardening fit. Constant-true-strain-rate loading was implemented by updating the crosshead velocity as the specimen height decreased. The control program calculated the instantaneous height and the corresponding velocity command from the prescribed rate and elapsed time, according to the following relations:

(4) $$v(t)={\dot{\varepsilon }}_{\mathrm{ref}}h(t)=h_{0}{\dot{\varepsilon }}_{\mathrm{ref}}\exp(-{\dot{\varepsilon }}_{\mathrm{ref}}t)$$

where *h*_0_ and *h*(t) were the initial and instantaneous specimen heights. *t* was time. ${{\dot{\varepsilon }}^{*}}_{\mathrm{ref}}$ was the prescribed positive true compressive strain rate and *v*(t) was the positive magnitude of the corresponding compressive crosshead speed. The sign convention therefore reported compression as a positive strain while *v*(t) denoted speed magnitude. The initial height was 5 mm. The calculated velocity history began at a crosshead-speed magnitude of approximately 0.3 mm/min and was applied through closed-loop displacement control. The measured displacement channel and the velocity command used the same initial-height definition.

The controller recalculated *h*(t) and *v*(t) as time advanced. The velocity command decreased with specimen height according to the kinematic relation, while the target value of ${\dot{\varepsilon }}_{\mathrm{ref}}$ remained fixed. At each acquisition step, the measured displacement and initial height defined the current specimen height used in true-strain processing. Machine control and data reduction therefore used one height history and one positive compression convention. Load and displacement were recorded synchronously with a load cell and a linear variable differential transformer. Engineering stress and engineering strain were calculated from the initial specimen dimensions. Engineering stress was converted to true stress using the following relation:

*σ_t_* = *σ_e_*(1 − *ε_e_*) (5)

Positive true compressive strain was then obtained from engineering strain using the following relation:

*ε_t_* = −ln(1 − *ε_e_*) (6)

where *σ*_e_ and *ε*_e_ denoted engineering stress and engineering strain, respectively. *σ*_t_ and *ε*_t_ denoted the corresponding true stress and positive true compressive strain, respectively. Both conversions used the plastic-incompressibility assumption. The initial linear part of each true stress–strain record supplied the apparent elastic modulus. To obtain equivalent plastic strain, elastic strain was removed by

*ε_p_* = *ε_t_* − *σ_t_*/*E* (7)

The flow stress at *ε*_p_ = 0.002 defined *A* = 907.95 MPa.

**Figure 1:**
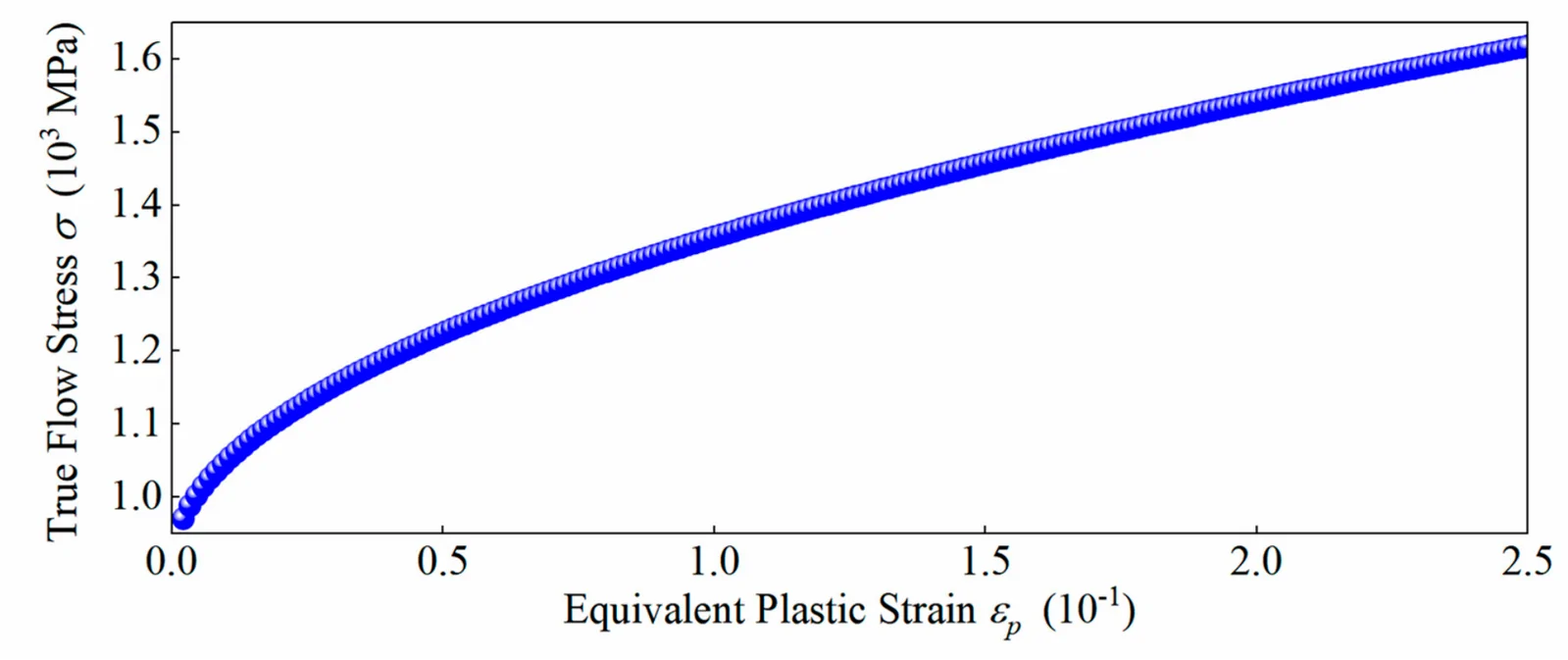
Johnson–Cook plasticity constitutive fitting curves for GH4096 superalloy at reference strain rate and reference temperature derived from specimen stress–strain relationships.

Engineering stress was obtained from the recorded load and initial cross-sectional area, and engineering strain from the recorded displacement and initial height. Conversion to true quantities preceded the elastic correction. The resulting equivalent plastic-strain coordinate was used both to define *A* and to arrange the data supplied to the *B* and *n* regression. At the reference rate and temperature, the rate and temperature multipliers take their reference values and the plasticity relation reduces to *σ* = *A* + *Bε*^n^. Subtracting *A* from the measured flow stress isolates the power-law hardening contribution. The fitted JC strain-hardening parameters are presented in Figure 1. Regression over the stable plastic-flow interval yielded *n* = 0.50 and *B* = 1421.96 MPa. Together with *A*, these values formed the reference hardening input used for the dynamic and temperature fits. The same transformation was applied to the processed compression curves. *A* remained fixed at the residual-strain value while *B* and *n* were obtained from the common linear form.

#### 2.1.3. High-Strain-Rate Identification of C and m

SHPB compression supplied the dynamic and heated data. Room-temperature impacts at 25 °C and nominal strain rates of 1000, 2000 and 3000 s^−1^ were used to identify *C*. Heated impacts at initial temperatures of 400, 600 and 800 °C were used to identify m after correction for deformation-generated heating. Dynamic compression specimens had dimensions Ø 4 mm × 3 mm. This geometry was used with the one-dimensional bar-wave treatment and the axial interface-force check. An annealed miniature copper pulse shaper was positioned at the center of the incident-bar impact face. Plastic deformation of the pulse shaper attenuated the high-frequency leading component and formed a smooth trapezoidal incident pulse with an extended rise. Semiconductor strain gauges recorded the incident, reflected and transmitted strain histories at a megahertz-scale sampling rate. The three histories were retained as separate channels through wave processing. The three recorded wave histories were first used to reconstruct the forces at the incident and transmitted specimen interfaces. Once their overlap established dynamic equilibrium, the equilibrium-reduced SHPB relations converted the transmitted history to specimen stress σ_s_(t) and the reflected history to positive specimen strain rate ${\dot{\varepsilon }}_{s}$(t) and specimen strain ε_s_(t). Specimen stress was reconstructed from the transmitted wave, which was given by

(8) $$\sigma_{s}(t)=\frac{A_{b}E_{b}}{A_{s}}\varepsilon_{T}(t)$$

Positive specimen strain was reconstructed by integrating the reflected wave, which was obtained by

(9) $$\varepsilon_{s}(t)=-\frac{2C_{0}}{L_{s}}\int_{0}^{t}\varepsilon_{r}(\tau )\mathrm{d\tau}$$

where the variables *A*_b_, *E*_b_ and *C*_0_ denoted the bar cross-sectional area, elastic modulus and longitudinal wave speed, respectively. *A*_s_ and *L*_s_ denoted the instantaneous specimen area and length used in the reconstruction, respectively. The histories *ε*_i_(t) and *ε*_r_(t) were the incident and reflected bar strains. The transmitted bar strain was the capital-T quantity in the stress relation. Although the incident history did not appear in either equilibrium-reduced relation, its superposition with the reflected history supplied the incident-interface force used in the equilibrium check. The strain-rate history was the time derivative of the reconstructed strain. A common bar-wave sign convention was applied before reconstruction, and the leading minus sign in the integrated strain relation mapped the reflected-wave sign to the positive specimen-compression convention. Records entered parameter fitting when the reconstructed interface forces overlapped through the main plastic-flow stage, the waveform remained smooth and the strain-rate history contained a stable interval. The equilibrium and stable-rate intervals defined the data used for regression at every nominal rate and temperature.

**Figure 2:**
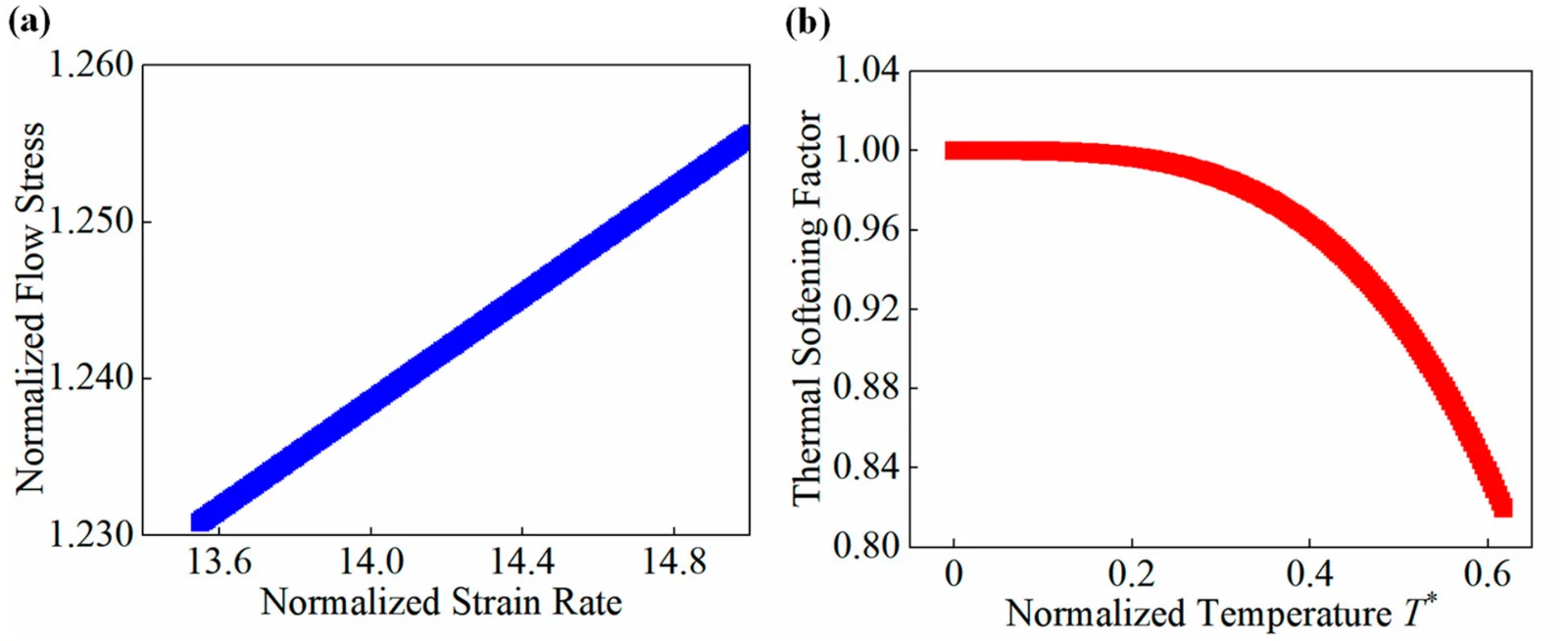
Calibration curves of Johnson–Cook plasticity parameters *C* and *m* based on the compression test data: (**a**) fitting curve of strain-rate coefficient *C*; (**b**) fitting curve of thermal softening exponent *m*.

The reconstructed stress and strain were expressed in the same positive compression convention used for the quasi-static curves. Alignment by equivalent plastic strain placed the room-temperature histories on the common deformation coordinate used in the *C* regression. At 25 °C, the temperature multiplier takes its reference value and the plasticity relation becomes *σ* = (*A* + *Bε*^n^)(1 + Cln($\dot{\varepsilon }$/${\dot{\varepsilon }}_{0}$)). Rearranging the equation gave *σ*/(*A* + *Bε*^n^) = (1 + Cln($\dot{\varepsilon }$/${\dot{\varepsilon }}_{0}$)). With *A*, *B* and *n* fixed, algebraic separation of the hardening contribution supplied the linear regression used to identify *C*. In this regression, ln($\dot{\varepsilon }$/${\dot{\varepsilon }}_{0}$) was the independent variable, and the transformed dynamic stress term was the dependent variable. The fitted curve was shown in Figure 2a. Room-temperature SHPB data at the three nominal rates entered one regression under the common reference state. The fitted slope gave *C* = 0.017.

The temperature rise generated by plastic work was evaluated incrementally before m was fitted. At increment k, the equivalent flow stress and equivalent plastic-strain increment supplied the plastic-work increment, while mass density and specific heat were evaluated at the current temperature. The Taylor–Quinney coefficient converted the corresponding fraction of plastic work to heat. After *A*, *B*, *n* and *C* had been fixed, the corrected instantaneous temperatures were used in the JC temperature term and its logarithmic regression. The expression of the curve after normalization was

(10) $$\frac{\sigma }{\left[A+B{\left({\bar{\varepsilon }}_{p}\right)}^{n}\right]\left[1+C\ln\left({\dot{\varepsilon }}_{p}/{\dot{\varepsilon }}_{0}\right)\right]}=1-{\left(\frac{T-T_{0}}{T_{m}-T_{0}}\right)}^{m}$$

The temperature was updated along the stress–strain history of each specimen, so every regression point contained its initial test temperature and its deformation-generated increment. The corrected temperature was converted to the same dimensionless temperature ratio used in the plasticity card. Data from the three heated groups were then combined under one constant value of *m*. The obtained normalized curve was shown in Figure 2b. At each increment, the current temperature was advanced by a plastic-work temperature increment evaluated from the current equivalent flow stress and equivalent plastic-strain increment. A Taylor–Quinney coefficient of 0.9 was used to convert plastic work to heat, while *ρ* and *C*_p_ supplied the mass density and specific heat at the current temperature. The updated temperature was converted to the dimensionless temperature ratio defined after Equation (1), using 25 °C and 1280 °C as the two temperature bounds. Corrected data from the 400, 600 and 800 °C groups entered one regression, giving the constant exponent *m* = 3.56. The completed plasticity card was then supplied to the non-isothermal cutting calculation.

The calibrated values were compared with published Inconel 718 and FGH96 parameter sets in Table 1. For the Inconel 718 parameter set [17], the reported transition and melting temperatures were 20 and 1260 °C, respectively, whereas the reference strain rate, detailed calibration range, and heat-treatment condition were not specified together with the parameter set. For FGH96 [26], the reference strain rate was 0.001 s^−1^, with *T*_0_ = 25 °C and *T*_m_ = 1350 °C; the parameters were obtained from SHPB tests at 4000–12,000 s^−1^ and 25–800 °C for HIP material. The FGH96 model adopted a modified Johnson–Cook (JC) formulation containing a strain-rate-dependent thermal exponent and an additional recrystallization-softening term, whereas the present GH4096 model employs a JC formulation with a constant thermal-softening exponent *m*.

The differences among the GH4096, Inconel 718, and FGH96 parameter sets are closely related to their distinct microstructures and thermomechanical histories. The higher *A* of GH4096 may be associated with stronger precipitation strengthening, grain refinement, and the high resistance to dislocation motion produced by HIP and subsequent isothermal forging. Its lower *n* suggests that a relatively high initial strength is already provided by fine strengthening precipitates, reducing the additional hardening generated by dislocation accumulation. Differences in *B* are mainly related to precipitate shearing or bypassing, dislocation storage, and dynamic recovery, which are strongly influenced by forging and heat-treatment conditions. The lower *C* of GH4096 relative to FGH96 may reflect differences in precipitate stability, dislocation mobility, and calibration strain-rate range. The marked variation in *m* is associated with high-temperature microstructural evolution, including precipitate coarsening or dissolution, dynamic recovery, and recrystallization.

### 2.2. Johnson–Cook Damage Model and Parameter Identification

#### 2.2.1. Johnson–Cook Damage Model

JC plasticity defined the deformation resistance, while the coupled damage model defined material-separation initiation and post-initiation evolution. At each material point, the cumulative initiation index was updated from the equivalent plastic-strain increment and the fracture strain under the current stress state, strain rate and temperature.

(11) $$D=\sum_{i}\frac{\Delta {\bar{\varepsilon }}_{i}^{\mathrm{pl}}}{{\bar{\varepsilon }}_{f}^{\mathrm{pl}}(\eta ,{\dot{\varepsilon }}^{*},T^{*})}$$

where *D* is the cumulative damage-initiation index. $\Delta {\bar{\varepsilon }}^{\mathrm{pl}}$ was the equivalent plastic-strain increment and $\Delta {\bar{\varepsilon }}_{f}^{\mathrm{pl}}$ was the fracture strain for the current state. *D* started from zero, and *D* = 1 activated the post-initiation degradation calculation. Lowercase *d* was reserved for the subsequent damage-evolution variable, whereas *D*_1_–*D*_5_ were reserved for the five JC fracture-strain coefficients.

At each increment, the current fracture strain was evaluated before the equivalent plastic-strain increment was added to *D*. This update connected the initiation history to the changing triaxiality, strain-rate ratio and temperature ratio at the same integration point. Once *D* reached unity, its role as the initiation index was complete and the subsequent stiffness update was carried out. The fracture strain was calculated from the JC damage relation, which was given by [27]:

(12) $${\bar{\varepsilon }}_{f}^{\mathrm{pl}}=\left[D_{1}+D_{2}\exp(D_{3}\eta )\right]\left[1+D_{4}\ln({\dot{\varepsilon }}^{*})\right]\left[1+D_{5}T^{*}\right]$$

where *η* was stress triaxiality, defined as the tension-positive ratio of mean stress to von Mises equivalent stress. ${\dot{\varepsilon }}^{*}$ used the same reference rate of 0.001 s^−1^ as the plasticity relation, and T* used *T*_0_ = 25 °C and *T*_m_ = 1280 °C. *D*_1_, *D*_2_ and *D*_3_ defined the triaxiality term. *D*_4_ defined the rate term and *D*_5_ defined the temperature term. All five coefficients were dimensionless. The local values of triaxiality, strain-rate ratio and temperature ratio were updated during the cutting calculation.

#### 2.2.2. Inverse Identification of D_1_–D_3_

*D*_1_, *D*_2_ and *D*_3_ were identified inversely from existing GH4096 notched-tension load–displacement records. The recorded histories, initial diameter, notch radius and minimum-section geometry supplied the inputs for deriving local fracture quantities. At 25 °C and the reference strain rate, the rate and temperature terms took their reference values, so the paired triaxiality and fracture-strain data defined the first damage term. The Bridgman relation supplied representative stress triaxiality η at the notched minimum section through the following relation, which was given by

(13) $$\eta =\frac{1}{3}+\ln\left(1+\frac{a}{2R}\right)$$

After introducing the stress triaxiality, if the conditions were set at the reference strain rate and reference temperature, the JC damage relation reduced to the following expression:

(14) $${\bar{\varepsilon }}_{f}=D_{1}+D_{2}\exp\left(D_{3}\eta \right)$$

**Figure 3:**
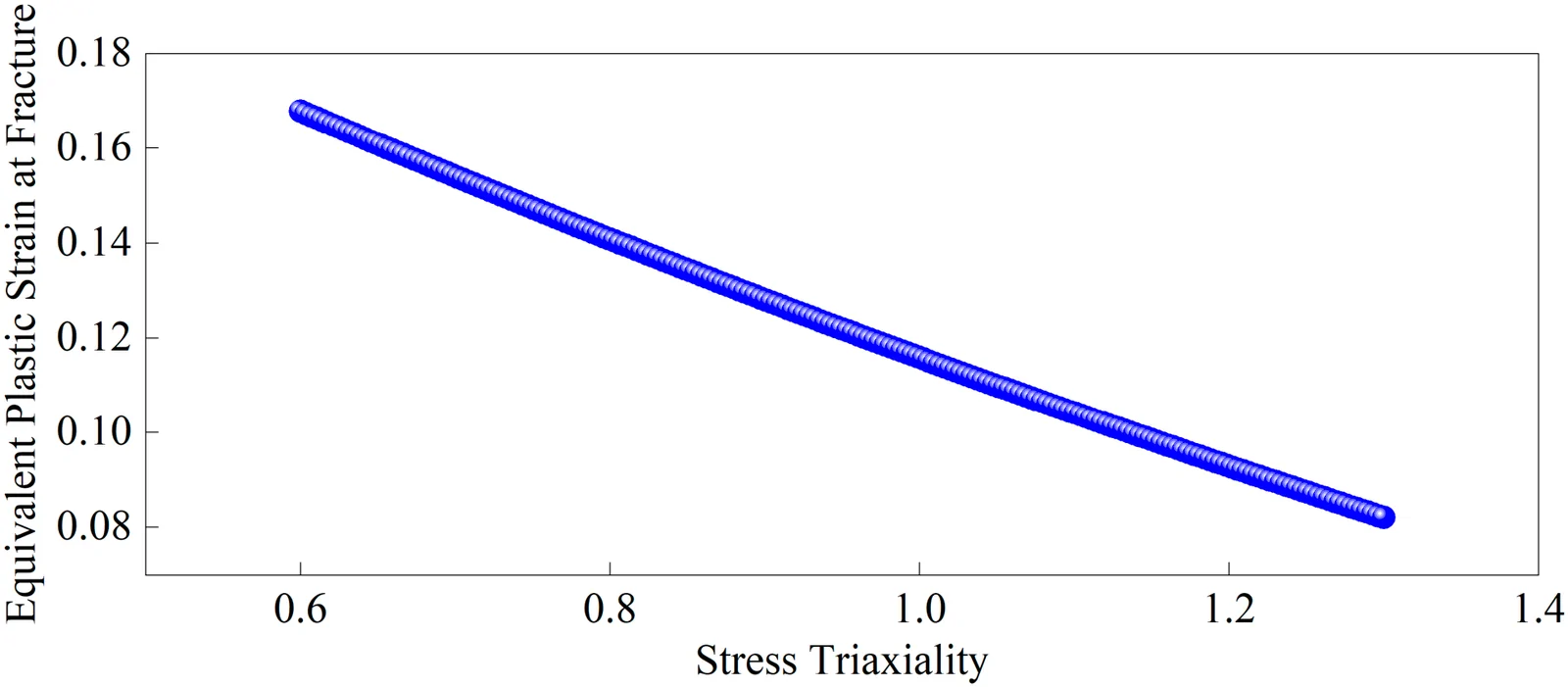
Joint fitting curves of fracture strain versus stress triaxiality for calibrating Johnson–Cook damage initiation parameters (*D*_1_–*D*_3_) of GH4096 superalloys.

In Equation (13), *a* was the minimum-section radius and *R* was the notch-curvature radius. Figure 3 shows the joint fit of the notched-tension fracture data under the tension-positive triaxiality convention. The representative triaxiality interval was approximately 0.60–1.30. The fitted values were *D*_1_ = −0.18, *D*_2_ = 0.44 and *D*_3_ = −0.41.

#### 2.2.3. Analysis and Identification of D_4_ and D_5_ from SHTB Experimental Data

*D*_4_ and *D*_5_ were obtained from GH4096 split Hopkinson tension bar (SHTB) experiments. Room-temperature tests at multiple strain-rate levels supplied the *D*_4_ data, whereas SHTB tests under multiple high-temperature conditions supplied the *D*_5_ data. The two groups were processed sequentially to isolate the strain-rate and temperature contributions. Incident, reflected and transmitted strain histories were synchronized to a common time origin and converted to a tension-positive sign convention. The one-dimensional-wave procedure used for the SHPB data in Section 2.1.3 was then reused to reconstruct dynamic tensile stress, strain rate and strain. Specimen-end force histories obtained from the incident-plus-reflected and transmitted signals were compared over the principal plastic-deformation interval. Only records showing dynamic force equilibrium and a smooth reconstructed response were retained; tests exhibiting grip slip, eccentric loading or fracture outside the effective gauge section were excluded. For each accepted record, the fracture instant was identified from the terminal load drop and the corresponding gauge-section deformation. Equivalent plastic fracture strain was extracted from the valid curve and corrected fracture cross-section consistently with the area-reduction treatment in Equation (14). Each value was paired with the representative stress triaxiality, dimensionless strain-rate ratio and temperature ratio of the same test to form the regression data. At room temperature, T* = 0, so the temperature multiplier in Equation (12) reduces to unity. With *D*_1_–*D*_3_ fixed, division by the triaxiality term isolated the rate-dependent part. Regression of the normalized fracture response against ln${\dot{\varepsilon }}^{*}$ therefore identified *D*_4_ from the slope of Equation (15), which was given by

(15) $$\frac{{\bar{\varepsilon }}_{f}^{\mathrm{pl}}}{D_{1}+D_{2}\exp(D_{3}\eta )}-1=D_{4}\ln{\dot{\varepsilon }}^{*}$$

For the high-temperature SHTB group, the imposed initial temperature was corrected for the adiabatic rise produced by plastic work using the Taylor–Quinney procedure described in Section 2.1.3. *D*_4_ was then held fixed, and the rate multiplier was divided out before the temperature regression. The corrected fracture response was fitted against T*, defined using *T*_0_ = 25 °C and *T*_m_ = 1280 °C; the slope of Equation (16) identified *D*_5_, which was obtained by

(16) $$\frac{{\bar{\varepsilon }}_{f}^{\mathrm{pl}}}{\left[D_{1}+D_{2}\exp(D_{3}\eta )][1+D_{4}\ln{\dot{\varepsilon }}^{*}\right]}-1=D_{5}T^{*}$$

The sequential fits gave *D*_4_ = 0.04 and *D*_5_ = 0.63.

The calibrated GH4096 damage parameters were compared with the Johnson–Cook damage parameters reported for Inconel 718 by Lu et al. in Table 2. The differences in damage parameters are closely related to the precipitate structures produced by different heat treatments. GH4096 is typically a sub-solvus solution treated and aged, retaining primary γ′ at grain boundaries while forming dense secondary and tertiary γ′ in the matrix. This stable γ′-strengthened structure limits local plastic accommodation under tensile triaxiality, consistent with the lower *D*_1_ and more negative *D*_3_. In contrast, aged Inconel 718 is mainly strengthened by γ″ and γ′; the lower thermal stability of γ″ makes its fracture response more temperature-sensitive, corresponding to the much larger *D*_5_. The lower *D*_4_ of GH4096 also indicates a weaker strain-rate dependence.

### 2.3. Finite Element Modeling of Laser-Assisted Milling of GH4096 Alloy

**Figure 4:**
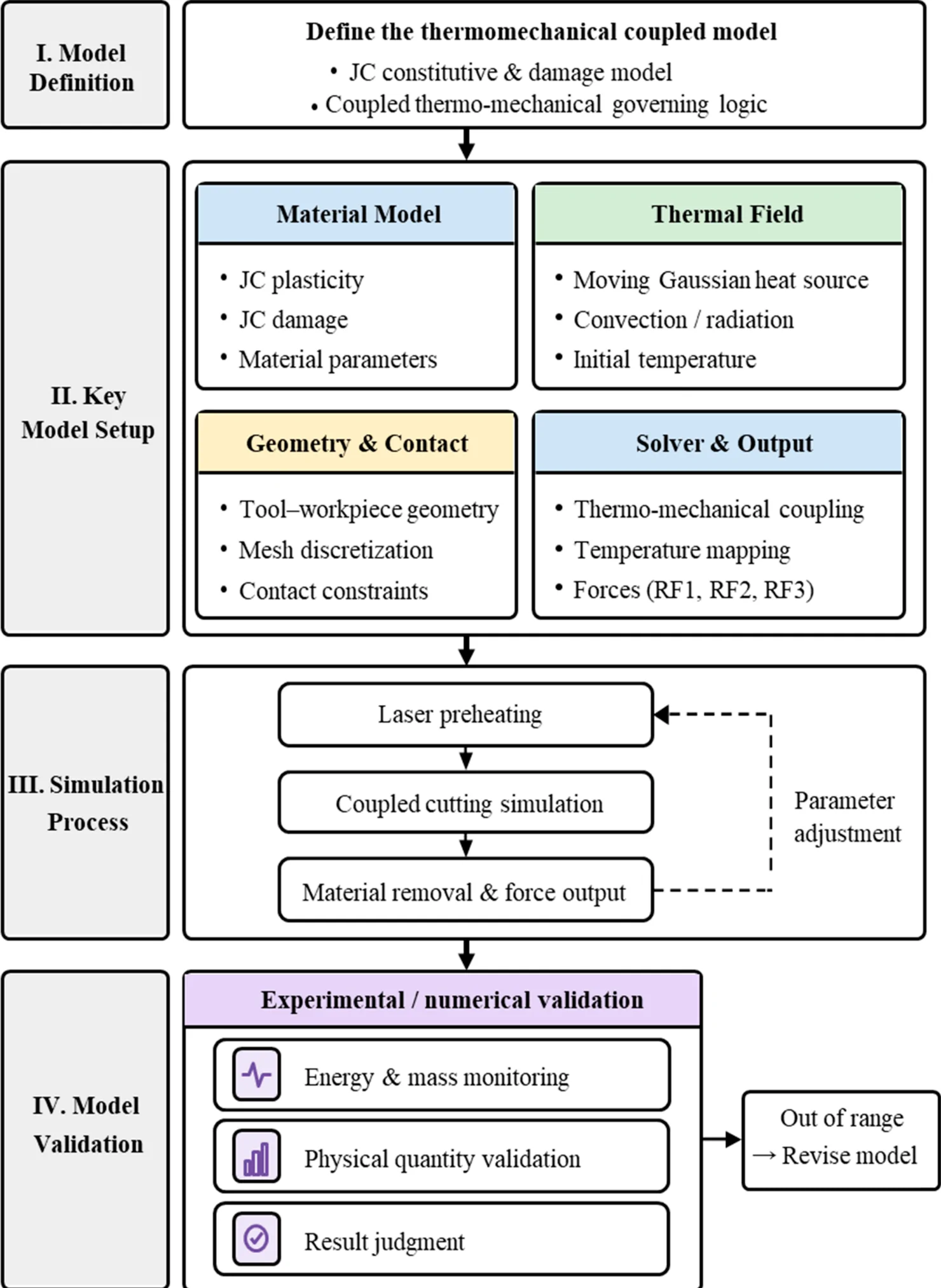
Workflow for the development and validation of the thermo-mechanically coupled numerical model for laser-assisted cutting, including model definition, key parameter setup, coupled cutting simulation, and model validation with parameter refinement.

The thermomechanical model of laser-assisted milling (LAM) incorporated Johnson–Cook (JC) plasticity, JC damage initiation and displacement-based linear damage evolution. Local plastic strain, strain rate, temperature and stress triaxiality governed the material response; D identified damage initiation, while d described subsequent degradation. The GH4096 density was 8000 kg/m^3^. The temperature-dependent property tables specified elastic modulus and Poisson’s ratio as 117.2 GPa and 0.14 at 21.1 °C, and 17.2 GPa and 0.22 at 1500 °C. Thermal conductivity was 50.2 W/(m·K) at 25 °C and 41.7 W/(m·K) at 1500 °C; specific heat was 391 J/(kg·K) at 25 °C and 525 J/(kg·K) at 800 –1500 °C. Thermal expansion coefficients were 9.1 × 10^−6^ K^−1^ at 20 °C and 1.05 × 10^−5^ K^−1^ at 1500 °C, referenced to 0 °C. Latent heat was 2.5 × 10^5^ J/kg, with solidus and liquidus temperatures of 1280 and 1350 °C [26]. The temperature-dependent thermal-property inputs were specified with reference to generic metal parameter settings and treated as modeling assumptions. Together with plastic-work conversion and interfacial frictional heating, detailed below, these thermal properties formed the basis of the coupled thermal response to laser irradiation and mechanical cutting. JC failure plastic displacement decreased from 0.006 mm at 400 °C to 0.0035 mm at 1500 °C. Additional shear damage used failure plastic strains of 0.35 and 0.001, with failure plastic displacements of 0.015 and 7.7 × 10^−3^ mm at 25 and 1500 °C, respectively. Damage contributions were combined with element deletion enabled and maximum degradation set to 0.85. Figure 4 summarizes the modeling workflow.

Laser heating was simulated using a moving Gaussian surface heat source with a laser–tool separation of 2 mm. Optical absorptivity was prescribed as *η*_abs_ = 0.40 with reference to generic metal absorptivity. A Fortran heat-flux subroutine updated the beam position with time and evaluated the absorbed surface flux according to Equation (17):

(17) $$q(x,y,t)=\frac{\eta_{\mathrm{abs}}\mathrm{Pcos\theta}}{\pi R^{2}}\exp\left[-\left(\frac{{\left(x-\mathrm{vt}\right)}^{2}}{{\left(R/\mathrm{cos\theta}\right)}^{2}}+\frac{y^{2}}{R^{2}}\right)\right]$$

**Figure 5:**
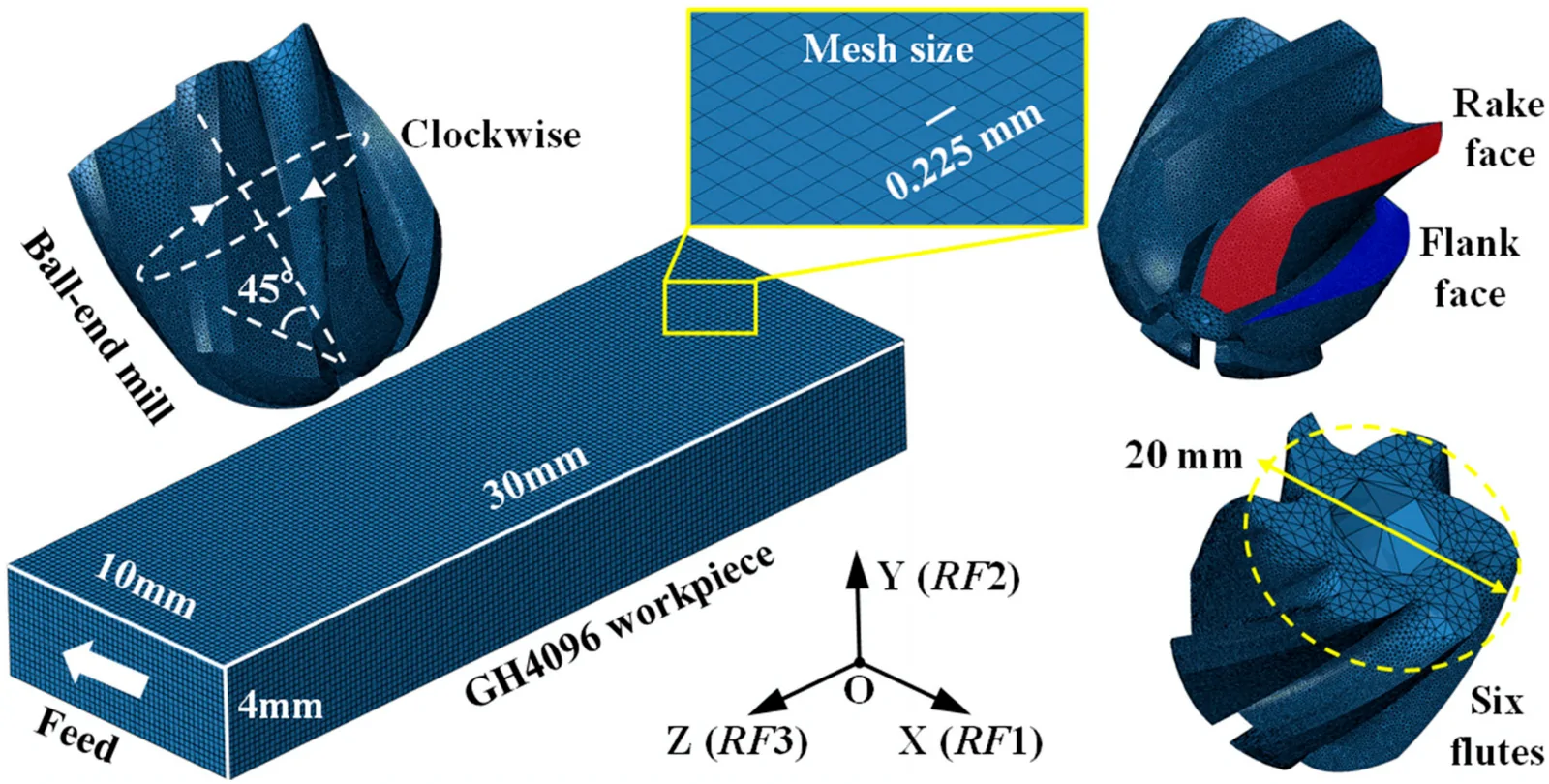
Numerical setup and assembly of the thermomechanical finite element model for laser-assisted ball-end milling simulation of GH4096 superalloy.

Tool–workpiece interaction used hard normal contact with separation permitted, penalty enforcement and finite sliding. Temperature-dependent Coulomb friction employed coefficients of 0.20 from 25 °C to 1500 °C [17], a shear-stress limit of 30 MPa and an elastic slip fraction of 0.005. The plastic-work and frictional-dissipation heat fractions were 0.90 and 1.00, respectively; all frictional heat was assigned to the workpiece. Contact conductance was zero at gap clearances of 0 and 0.001 mm, and the tool was constrained as an isothermal rigid body at 25 °C. Laser absorption, plastic dissipation, frictional heating and environmental heat exchange determined the thermal response, which updated JC flow stress and damage. Element deletion represented material separation and boundary evolution. Figure 5 shows the model assembly. All subsequent simulation images were captured in an isometric view from the positive X, Y and Z side. To highlight the primary deformation zone and improve the clarity of the multi-panel figures, only the machining region is displayed; the remaining workpiece regions with negligible deformation and the tool modeled as an isothermal rigid body are omitted.

An explicit coupled temperature–displacement analysis was performed with geometric nonlinearity and a cutting-step duration of 0.020 s. The workpiece used eight-node reduced-integration coupled elements with a global mesh seed size of 0.225 mm. Enhanced hourglass and distortion controls were enabled; linear and quadratic bulk-viscosity coefficients were 0.06 and 1.20. Automatic incrementation used a scaling factor of 1.00, with variable mass scaling targeting 5.0 × 10^−7^ s, and updated every increment. Field output comprised 768 intervals, while history output used an interval of 2.60417 × 10^−5^ s. *RF1*, *RF2* and *RF3* at the tool reference point represented the three force components acting on the tool along global X, Y and Z under the adopted reporting convention. Positive Y was upward; cutting the material beneath the tool therefore produced positive *RF2*. For the clockwise tool rotation viewed from above in the present setup, material-removal resistance produced negative *RF3*. Table 3 presents five parameter series, each with six levels, sharing a baseline of 300 W laser power, 2.0 mm spot radius, 0.6 mm cutting depth, 15 mm/s feed speed and 9000 r/min spindle speed.

### 2.4. Evaluation Metrics for Milling Force, Temperature Field, and Surface Von Mises Equivalent Stress

The responses discussed in Section 3 are characterized using the metrics defined below. The representative milling-force level, *F*_90_, is defined as the 90th percentile of the pooled absolute peak values of the feed, radial, and axial force components:

*F*_90_ = *Q*_0.90_(𝒫). (18)

Here, 𝒫 contains the magnitudes of positive peaks and negative troughs pooled from the three force components, and *Q*_0.90_ denotes the 90th percentile. *F*_90_ characterizes a representative high force level while reducing the influence of isolated extreme values; its unit is N.

A supplementary temperature statistic, *T*_90_, is also reported in Section 3. It denotes the 90th percentile of nodal temperatures within the selected region at the observation time specified for each comparison; its unit is °C.

*T*_p_. The maximum temperature within the evaluated region over the observation period is defined as

*T_p_* = max**_x_**_∈Ω,*t*∈𝒯_*T*(**x**,*t*). (19)

Here, *T*(**x**,*t*) is the temperature at position **x** and time *t*, Ω is the evaluated region, and 𝒯 is the set of recorded times. *T*_p_ characterizes the highest local thermal level; its unit is °C. For depth-resolved temperature analysis, the baseline mesh was divided into surface (Y = 0), subsurface (Y = −0.222 to −0.444 mm), and internal (Y = −0.667 to −1.778 mm) groups using the original global coordinates. These fixed layers describe thermal sampling locations rather than temperature-threshold-based preheating depths. Within each parameter series, comparisons retained the baseline grouping on common nodes; trajectory-based spatial bins and independently selected representative regions did not necessarily correspond to identical machine-coordinate locations across cases.

Mean temperature in the common region *T*_c_. The mean of all valid temperature samples within the region shared by the compared cases over the observation period is defined as

(20) $$T_{c}=\frac{1}{N}\sum_{n=1}^{N}T_{n}$$

Here, *T*_n_ is a valid temperature sample in the common region, and *N* is the total number of these samples across space and recorded time. *T*_c_ characterizes the overall thermal level on a common spatial basis; its unit is °C.

Temperature-rise fraction, *p*_50_. The fraction of samples in the common region with a temperature rise of at least 50 °C is defined as

(21) $$p_{50}=\frac{N_{50}}{N}.$$

Here, *N*_50_ is the number of samples satisfying $T_{n}-T_{0}\geq 50$ °C, and *T*_0_ is the reference temperature. *p*_50_ describes the prevalence of this temperature rise across the sampled space and time. It ranges from 0 to 1 and can be expressed as a percentage by multiplying by 100%.

Thermal-softening factor, *S*_T_. The temperature-dependent multiplier in the Johnson–Cook model is defined as

(22) $$S_{T}(T)=1-{\left(\frac{T-T_{0}}{T_{m}-T_{0}}\right)}^{m},\;\;T_{0}\leq T\leq T_{m}$$

Here, *T* is the evaluation temperature, *T_m_* is the melting temperature, and *m* is the thermal-softening exponent. *S*_T_ is dimensionless; a smaller value indicates stronger thermal softening at the same strain and strain rate. Substituting *T* = *T*_p_ gives the factor at the peak temperature.

Maximum surface equivalent stress, *σ*_s,max_. The maximum von Mises equivalent stress on the machined surface at the final recorded time is defined as

(23) $$\sigma_{s,\max}=\max_{x\in \Omega_{s}}\sigma_{\mathrm{vM}}(x,t_{\mathrm{end}})$$

Here, Ω*_s_* is the machined surface, *σ*_vM_ is the von Mises equivalent stress, and *t*_end_ is the final recorded time. *σ*_s,max_ characterizes the maximum surface equivalent-stress level in MPa. When the final state corresponds to unloading and cooling, it represents the maximum surface von Mises equivalent stress; it does not distinguish tensile from compressive stress.

### 2.5. Validation and Comparative Analysis of Laser-Assisted Milling

To validate the laser-assisted milling simulation model and assess surface quality, ball-end slot-milling experiments were conducted using a ball-end mill inclined at 45°. Milling forces were measured using a rotary dynamometer, and temperatures were recorded for comparison with the simulation results. White-light interferometry was used to characterize the machined surface topography and measure surface roughness. A continuous-wave laser with a wavelength of 1064 nm was used, with the spot radius adjusted to 2.0 mm. The simulations and experiments shared identical baseline parameters: laser power of 300 W, cutting depth of 0.6 mm, feed speed of 15 mm/s, and spindle speed of 9000 r/min. For the force comparison, both datasets were evaluated over the first 0.02 s of the force-bearing interval using the previously defined peak-extraction procedure.

**Figure 6:**
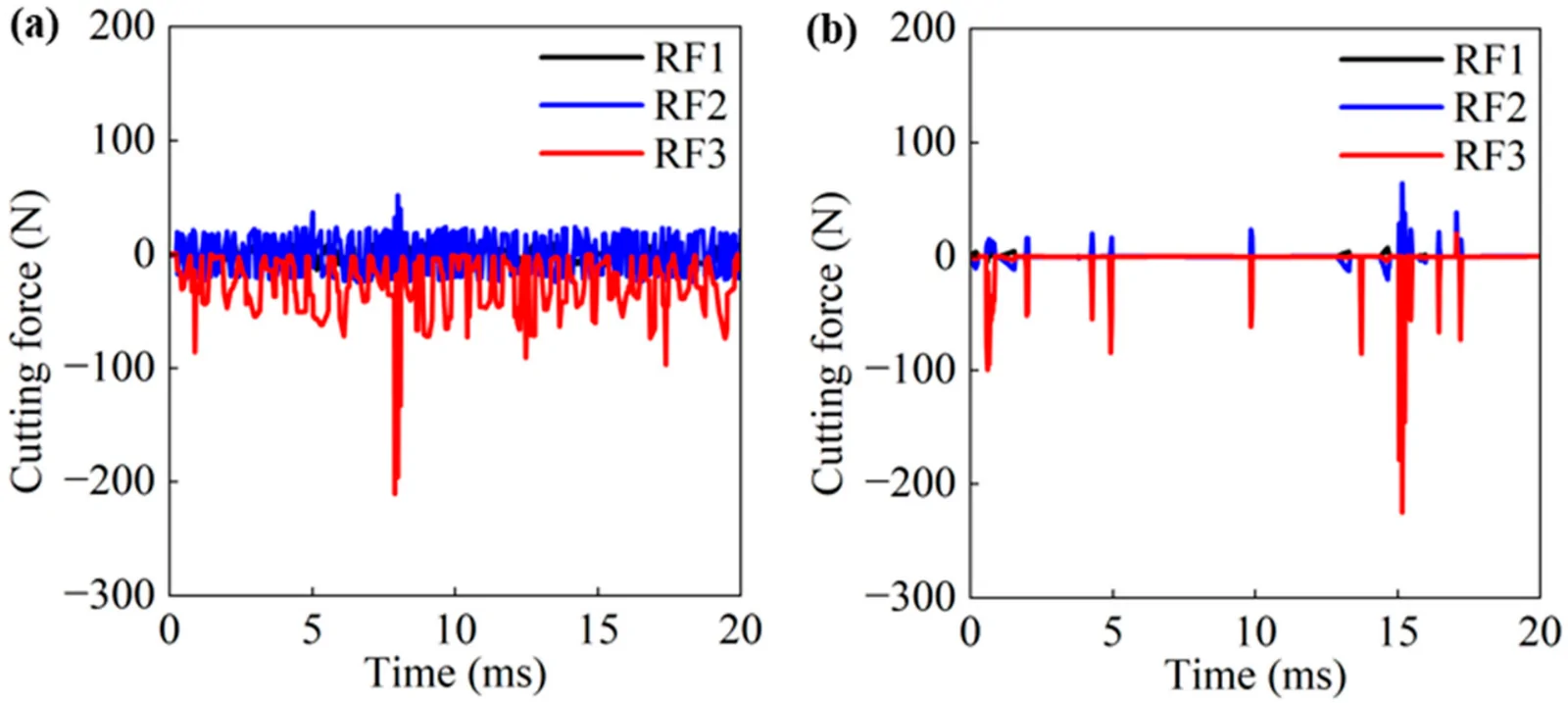
Comparison of experimental and simulated cutting forces in laser-assisted milling: (**a**) experimental results; (**b**) simulation results.

Figure 6 compares the experimental and simulated milling-force waveforms for laser-assisted milling. The experimental and simulated *F*_90_ were 11.12/11.62 N for *RF1*, 23.36/23.21 N for *RF2*, and 92.95/91.81 N for *RF3*, with relative errors of 4.45%, 0.60%, and 1.23%, respectively, using experimental values as the reference. The pooled *F*_90_ values were 47.64/50.11 N, yielding an error of 5.18%, below 15%. Both datasets followed *RF3* > *RF2* > *RF1*. Experimental and simulated maximum absolute forces were 16.28/16.87, 51.99/64.30, and 210.16/224.67 N, with errors of 3.58%, 23.68%, and 6.90%, respectively. Agreement in the dominant *RF3* component supports the predicted principal loading response. *RF2*’s *F*_90_ error, compared with its maximum-force error, demonstrates closer agreement in representative high-force levels. These results support the model’s prediction of characteristic force magnitudes and directional loading distribution.

The sustained experimental force reflects continuous milling during acquisition. Differences in sampling points and practical synchronization, including manual laser triggering, can contribute to differences in waveform continuity and peak timing. Evaluating peak statistics over the same duration accommodates temporal offsets while retaining the force amplitudes relevant to cutting loads. Together, the directional and pooled comparisons provide direct quantitative validation of the model’s milling-force predictions and support its use in analyzing the mechanical response of laser-assisted milling.

**Figure 7:**
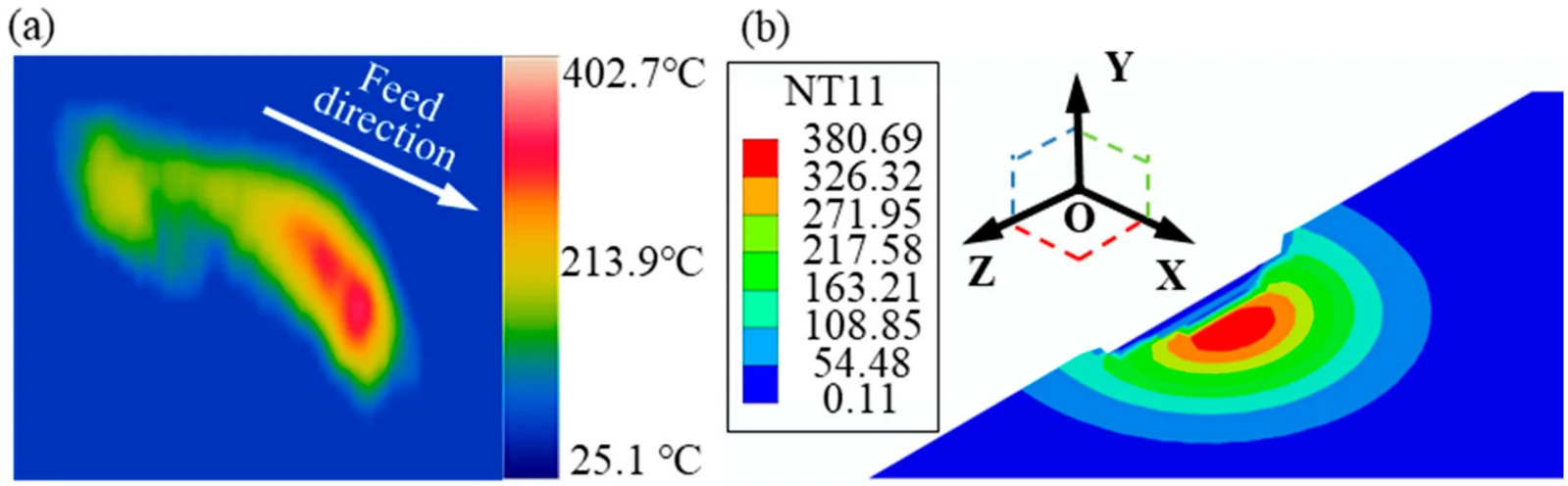
Comparison of experimental and simulated temperature fields in laser-assisted milling: (**a**) experimental temperature field; (**b**) simulated temperature field.

The experimental temperature field was measured using an Optris Xi 400 thermal imaging camera (Optris GmbH & Co. KG, Berlin, Germany). Figure 7 compares this field with the simulated temperature field under the same baseline conditions. The experimental and simulated maximum temperatures were 402.7 °C and 380.69 °C, respectively, giving an absolute difference of 22.01 °C and a relative error of 5.47% referenced to the experimental value. This close agreement provides a quantitative experimental benchmark for the model’s prediction of the peak thermal response.

Both temperature fields exhibit a localized high-temperature region surrounded by progressively cooler material, indicating that the simulation reproduces the principal spatial characteristics of laser-induced heating. The experimental distribution is more elongated and asymmetric, whereas the simulated contours are smoother. This difference is consistent with the inclined laser irradiation in the experiment, which modifies the projected heating footprint and contributes to the observed spatial asymmetry.

The agreement in peak temperature and the temperature-distribution pattern supports the material thermal-property settings adopted in the model, particularly their combined representation of heat storage and conduction during laser-assisted milling. Together with the milling-force comparison, these temperature results provide experimental evidence for the predictive capability of the thermomechanically coupled model.

**Figure 8:**
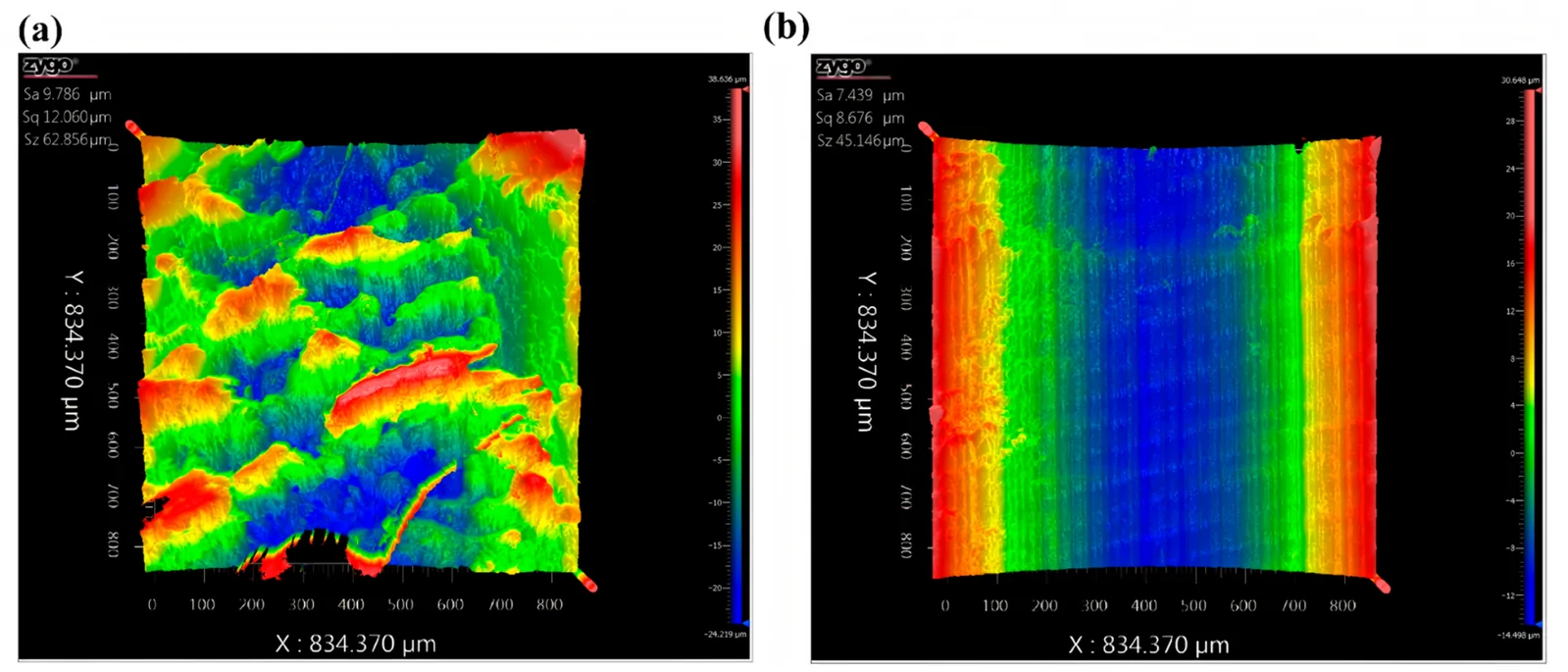
Surface topographies measured by white light interferometry under different milling conditions: (**a**) conventional milling; (**b**) laser-assisted milling.

Surface quality was further evaluated under the baseline settings, with the laser disabled for conventional milling. Figure 8 presents two groups of white-light interferometry topographies, each covering the front, middle, and rear positions of a single slot. The corresponding roughness (*Ra*)values were 9.786, 10.650, and 7.883 μm for conventional milling and 7.373, 7.439, and 6.603 μm for laser-assisted milling (LAM). The mean roughness (*Ra*) decreased from 9.44 ± 1.42 μm to 7.14 ± 0.46 μm, representing a 24.4% reduction. Values are reported as mean ± standard deviation across the three measurement positions, reflecting within-slot variability. The LAM surface also exhibited a smoother, more uniform topography. Together, the force and temperature comparisons support the model’s validity under the tested conditions, while the surface measurements demonstrate improved surface quality with laser assistance in the examined slots.

## 3. Process-Parameter Effects on Milling Force and Surface Von Mises Equivalent Stress in GH4096 Laser-Assisted Milling

### 3.1. Laser Power

**Figure 9:**
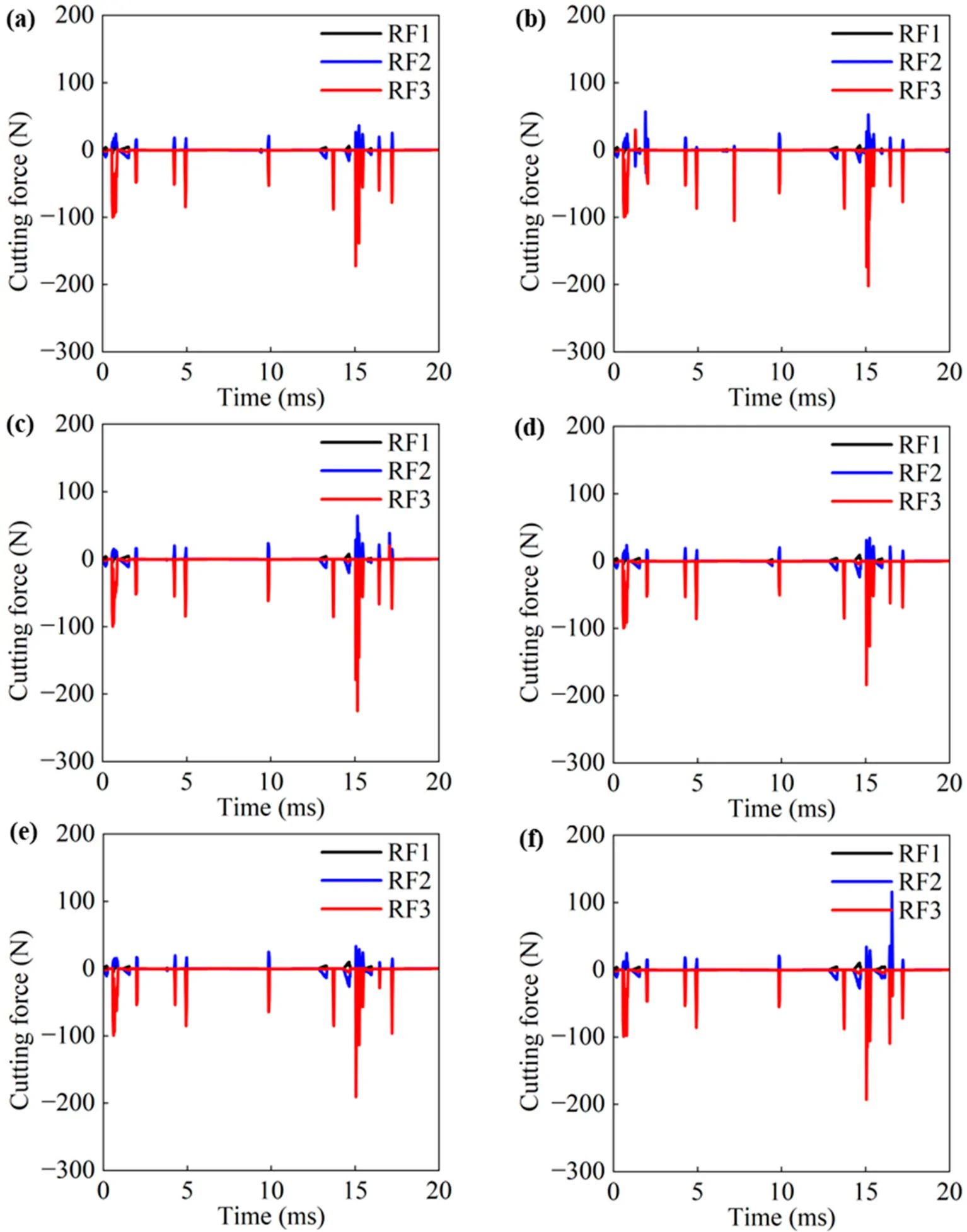
Time histories of the three milling-force components (*RF1*, *RF2*, and *RF3*) during laser-assisted milling of GH4096 under different laser powers: (**a**) 200 W, (**b**) 250 W, (**c**) 300 W, (**d**) 350 W, (**e**) 400 W, and (**f**) 450 W.

Figure 9 presents the milling-force curves obtained at laser powers of 200, 250, 300, 350, 400, and 450 W in panels (a)–(f), respectively. *RF3* is the dominant negative-direction impact component throughout the power series, and its larger negative peaks occur mainly during concentrated tooth engagement. *RF1* remains close to zero with limited fluctuation, whereas *RF2* is characterized by positive pulses and develops a pronounced late-stage positive peak at 450 W. Using the representative strong-peak metric *F*_90_ defined in Section 2.5, the values at 300, 400, and 450 W are 50.11, 35.20, and 47.23 N, respectively. The decrease from 300 to 400 W is 14.91 N, corresponding to 29.75%; the subsequent increase from 400 to 450 W is 12.03 N, corresponding to 34.18%. The 450 W value is 2.88 N lower than the 300 W value, with a difference of 5.75%. These values establish a decrease–rebound pattern in *F*_90_, with the minimum value at 400 W and the rebound at 450 W.

**Figure 10:**
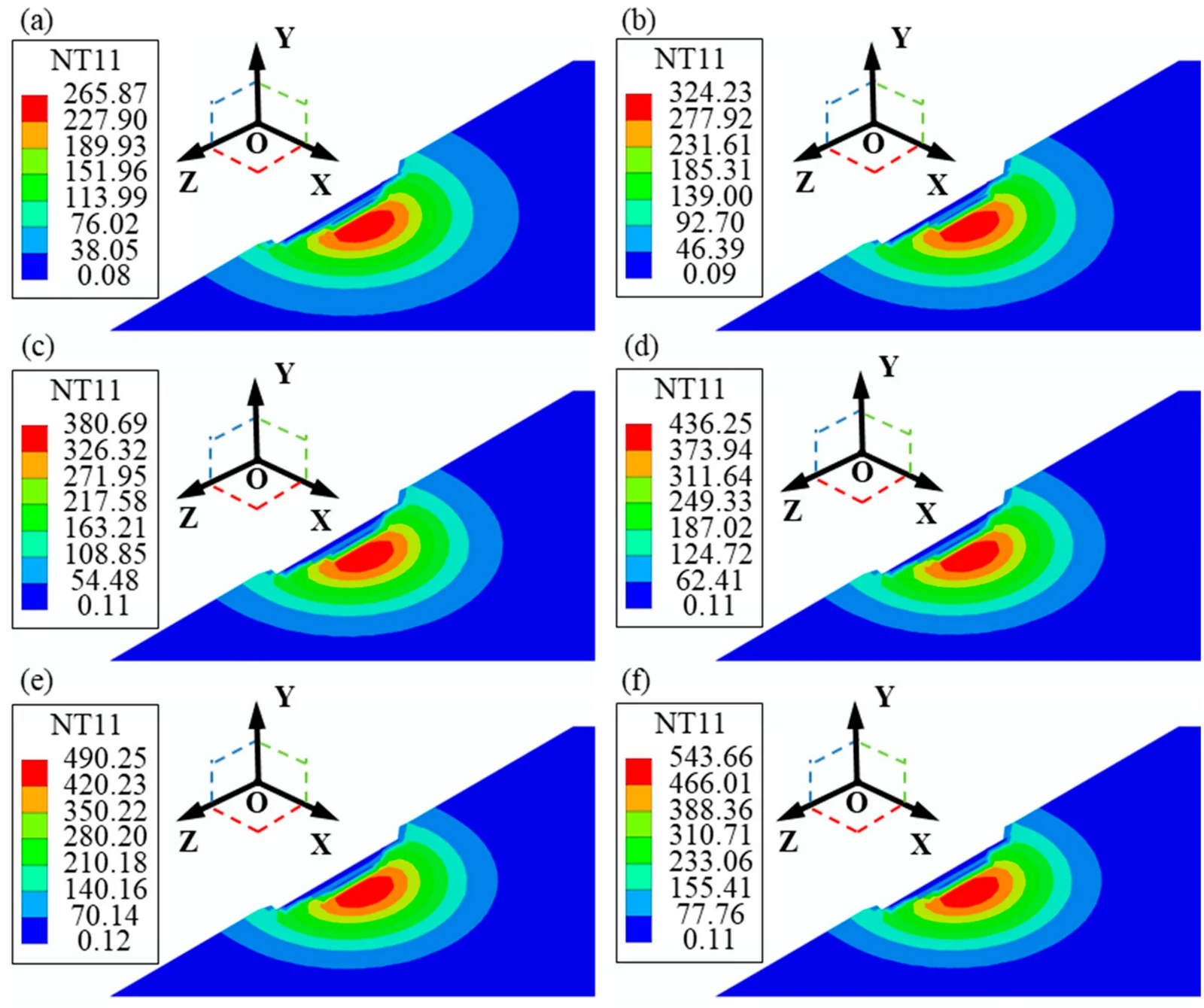
Temperature distributions during laser-assisted milling of GH4096 under different laser powers: (**a**) 200 W, (**b**) 250 W, (**c**) 300 W, (**d**) 350 W, (**e**) 400 W, and (**f**) 450 W.

Figure 10 shows that the temperature field increases continuously across the laser-power series. The peak temperature *T*_p_ rises from 498.12 °C at 200 W to 647.21 °C at 450 W, giving an increase of 149.09 °C, or 29.93%, equivalent to approximately 59.64 °C per 100 W. The mean temperature increases from 47.79 to 62.52 °C, an increase of 14.73 °C, or 30.82%, equivalent to 5.89 °C per 100 W. In the common 2659-node region, *T*_c_ increases from 38.17 to 53.24 °C, giving an increase of 15.07 °C, or 39.48%, equivalent to 6.03 °C per 100 W. Thus, the fixed-node-set temperature accumulation shows a stronger relative increase than the final-frame regional mean.

The proportion *p*_50_ increases from 7.25% at 200 W to 15.72% at 450 W. The absolute increase is 8.47 percentage points, corresponding to a relative increase of 116.83%, or approximately 3.39 percentage points per 100 W. Substitution of the two sampled peak temperatures into the *S_T_* relation defined in Section 2.5 gives *S*_T_ = 0.9690 and *S*_T_ = 0.9177, respectively. The corresponding change is 0.0513, or 5.29%, and it accompanies the 149.09 °C increase in *T*_p_. The temperature-related indicators therefore show a consistent power-dependent thermal intensification from 200 to 450 W.

The force and temperature series exhibit different response paths: *T*_p_, the final-frame regional mean, *T*_c_, and *p*_50_ increase continuously with laser power, whereas *F*_90_ reaches 35.20 N at 400 W and rebounds to 47.23 N at 450 W. The 450 W force level is therefore 34.18% higher than the 400 W level while the peak temperature continues to increase. This paired quantitative response describes the force–temperature evolution across the parameter series and provides the basis for relating the milling-force peaks to the thermal state at each power level.

**Figure 11:**
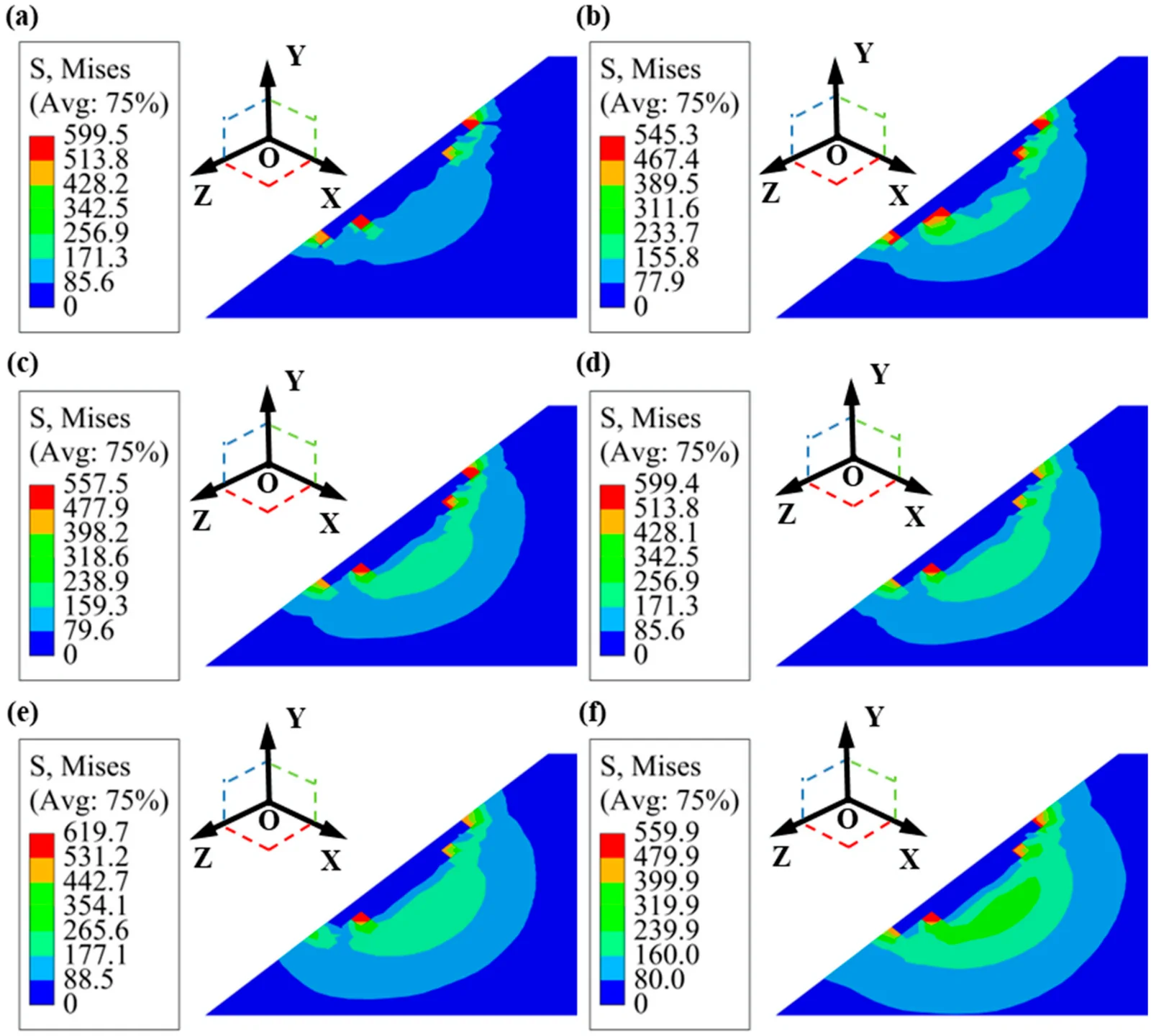
Von Mises stress distributions in the workpiece during laser-assisted milling of GH4096 under different laser powers: (**a**) 200 W, (**b**) 250 W, (**c**) 300 W, (**d**) 350 W, (**e**) 400 W, and (**f**) 450 W.

Figure 11 presents the machined-surface von Mises equivalent stress distributions. The maximum surface von Mises equivalent stress, denoted by *σ*_s,max_, is 599.45, 545.33, 557.54, 599.39, 619.75, and 559.88 MPa at 200, 250, 300, 350, 400, and 450 W, respectively. From 200 to 250 W, *σ*_s,max_ decreases by 54.12 MPa, or 9.03%. It then increases by 12.21 MPa from 250 to 300 W, by 41.85 MPa from 300 to 350 W, and by 20.36 MPa from 350 to 400 W. The value at 400 W reaches the series maximum of 619.75 MPa, which is 74.42 MPa, or 13.65%, higher than the minimum value of 545.33 MPa at 250 W. From 400 to 450 W, *σ*_s,max_ decreases by 59.87 MPa, or 9.66%, to 559.88 MPa. Across the full 200–450 W range, the endpoint difference is 39.57 MPa, corresponding to 6.60% relative to the 200 W value.

The spatial distributions follow the same staged evolution. At 200 W, the surface field is dominated by a relatively narrow blue–cyan band. With increasing power, the green gradient region becomes more continuous and wider at 350–400 W, while the local high-stress zones expand. At 400 W, this spatial development coincides with the maximum *σ*_s,max_. At 450 W, the affected region remains broad and the peak value changes to 559.88 MPa. The equivalent stress response consequently forms a decrease–increase–decrease sequence over the six power levels, with its maximum at 400 W.

The temperature and equivalent stress results together show a coordinated but differentiated parameter response. The peak temperature increases by 29.93% from 200 to 450 W, while *σ*_s,max_ varies through a 74.42 MPa range and reaches its maximum at 400 W. The 400 W condition combines a 619.75 MPa maximum surface von Mises equivalent stress with a broad high-stress affected region, whereas the 450 W condition retains a broad affected region with a lower peak of 559.88 MPa. These numerical and morphological features characterize the laser-power dependence of the machined-surface von Mises equivalent stress field.

### 3.2. Laser Spot Radius

**Figure 12:**
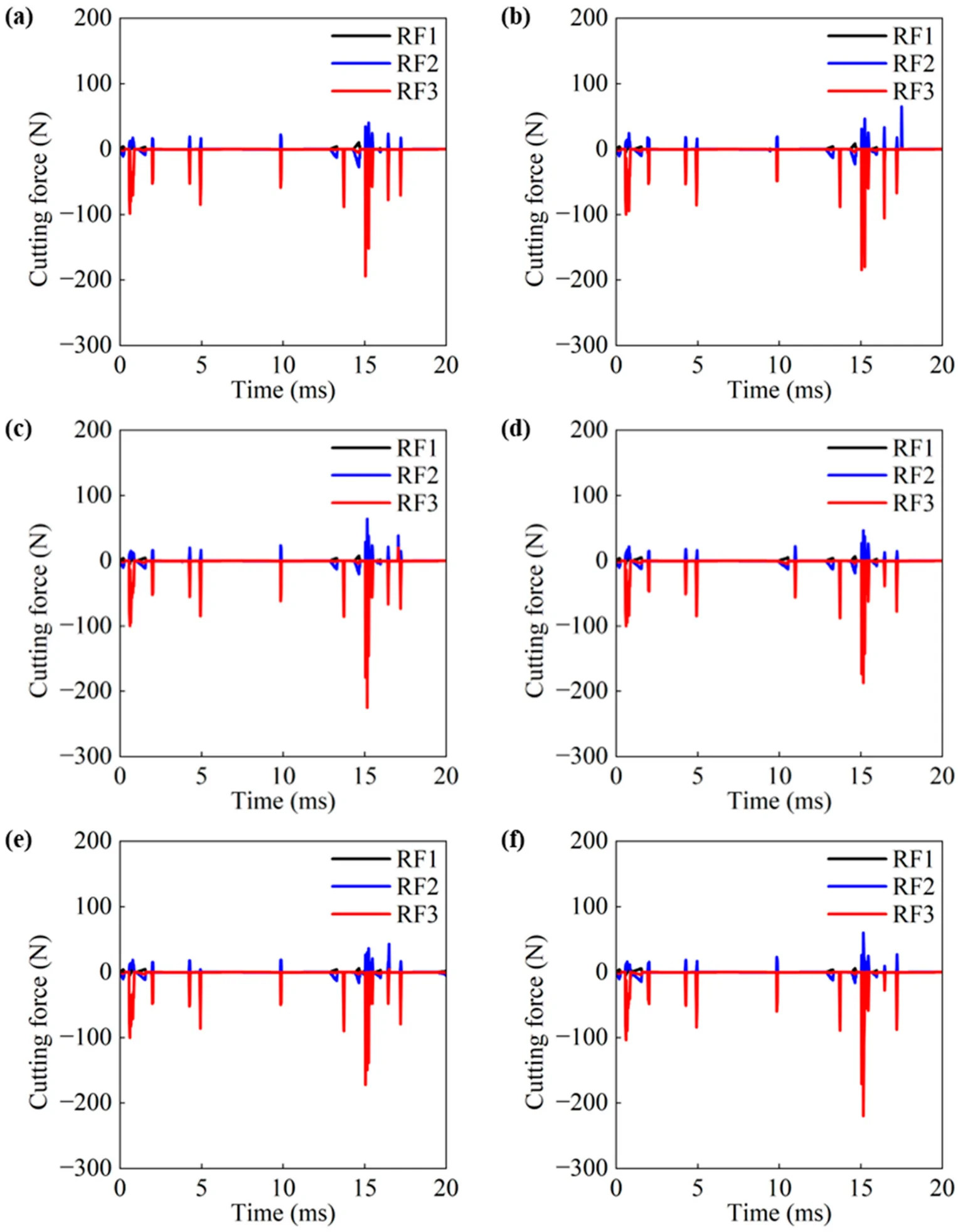
Time histories of the three milling-force components (*RF1*, *RF2*, and *RF3*) during laser-assisted milling of GH4096 under different laser spot radii: (**a**) 1.6 mm, (**b**) 1.8 mm, (**c**) 2.0 mm, (**d**) 2.2 mm, (**e**) 2.4 mm, and (**f**) 2.6 mm.

Figure 12 presents the milling-force curves obtained at laser spot radii of 1.6, 1.8, 2.0, 2.2, 2.4, and 2.6 mm in panels (a)–(f), respectively. *RF3* is the dominant impact component with negative values throughout the radius series, and its larger negative pulses occur mainly during concentrated tooth engagement. *RF1* remains comparatively small, whereas *RF2* is characterized mainly by positive peaks. Using the representative strong-peak metric *F*_90_ defined in Section 2.5, the values at 1.6, 2.4, and 2.6 mm are 52.27, 37.83, and 40.88 N, respectively. From 1.6 to 2.4 mm, *F*_90_ decreases by 14.44 N, corresponding to 27.63%; from 2.4 to 2.6 mm, it increases by 3.05 N, corresponding to 8.06%. The value at 2.6 mm remains 11.39 N, or 21.79%, below the value at 1.6 mm. These values establish a decrease–rebound pattern in *F*_90_, with the minimum value at 2.4 mm and a modest rebound at 2.6 mm.

**Figure 13:**
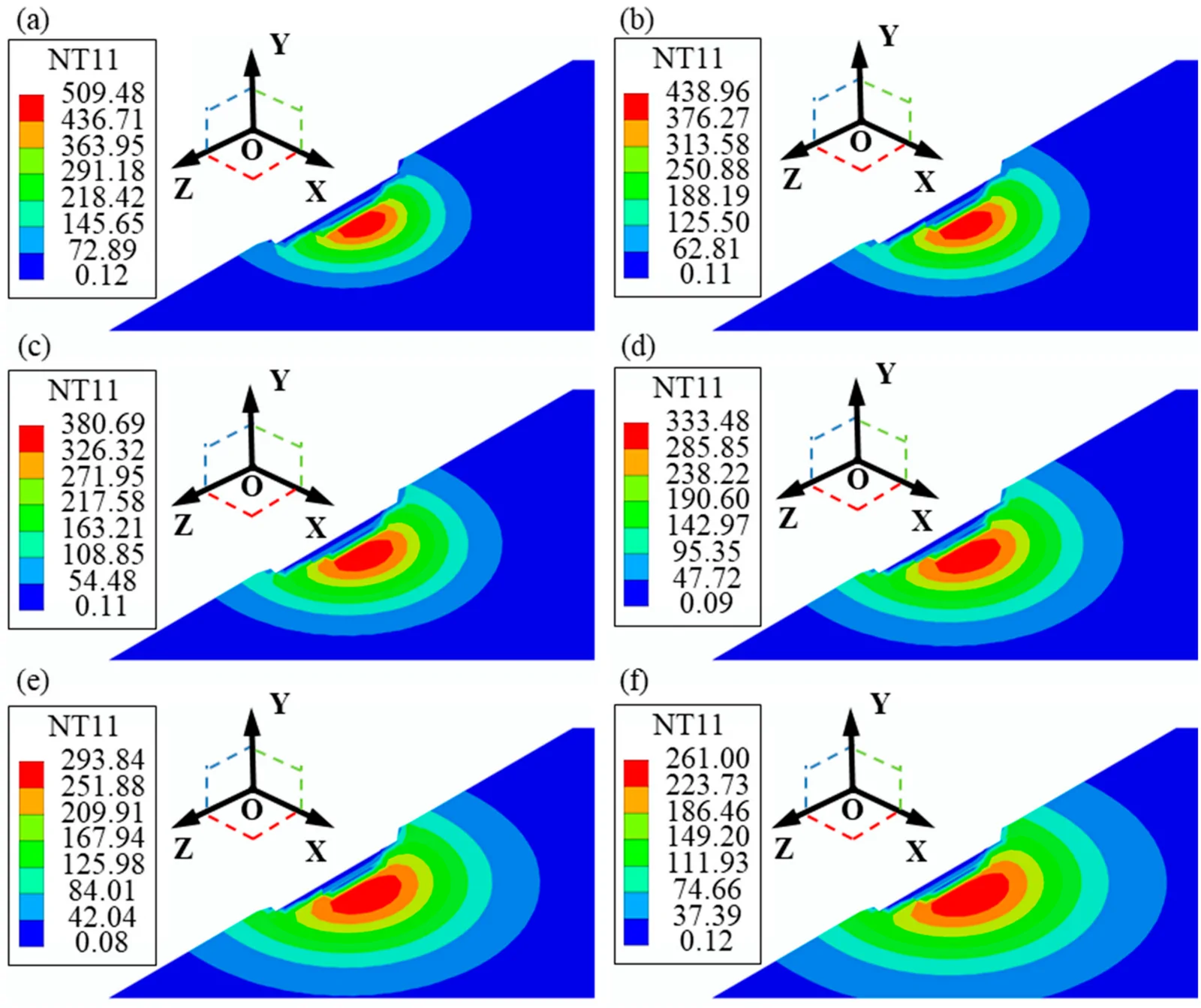
Temperature distributions during laser-assisted milling of GH4096 under different laser spot radii: (**a**) 1.6 mm, (**b**) 1.8 mm, (**c**) 2.0 mm, (**d**) 2.2 mm, (**e**) 2.4 mm, and (**f**) 2.6 mm.

Figure 13 shows the redistribution of the temperature field as the laser radius changes. At fixed laser power and feed speed, increasing the spot radius redistributes the nominal Gaussian input over a wider area rather than reducing the prescribed total power. The mean temperature decreases from 57.39 °C at 1.6 mm to 49.63 °C at 2.6 mm, a decrease of 7.76 °C, or 13.52%. The *T*_90_ decreases from 166.41 to 122.37 °C, a decrease of 44.04 °C, or 26.46%, indicating a stronger reduction in the upper-temperature tail than in the regional mean.

In the common 2524-node region, *T*_c_ increases from 44.49 °C at 1.6 mm to 45.35 °C at 2.2 mm, an increase of 0.86 °C, or 1.93%, and then decreases to 44.98 °C at 2.6 mm. The case-specific final-frame mean and *T*_90_ use independently selected regions, whereas *T*_c_ is the full-window mean over a common 2524-node region; the two summaries therefore differ in both spatial selection and temporal aggregation. The endpoint value at 2.6 mm remains 0.49 °C, or 1.10%, above the value at 1.6 mm. Thus, the full-window common-region mean follows a shallow rise–fall sequence, with a 0.37 °C decrease from 2.2 to 2.6 mm. The peak temperature *T*_p_ also follows a non-monotonic response and reaches 570.42 °C at 2.6 mm. These indicators show that spot radius changes the regional mean, upper-temperature tail, common-region accumulation, and local peak temperature with different response amplitudes.

The force and temperature responses develop along differentiated paths. *F*_90_ decreases from 52.27 N at 1.6 mm to 37.83 N at 2.4 mm and then rebounds to 40.88 N at 2.6 mm. Over the same endpoint interval, the final-frame regional mean and *T*_90_ decrease by 13.52% and 26.46%, whereas the common-region *T*_c_ increases slightly by 1.10%. This paired response describes the radius-dependent redistribution of the thermal field alongside the decrease–rebound evolution of the milling-force level.

**Figure 14:**
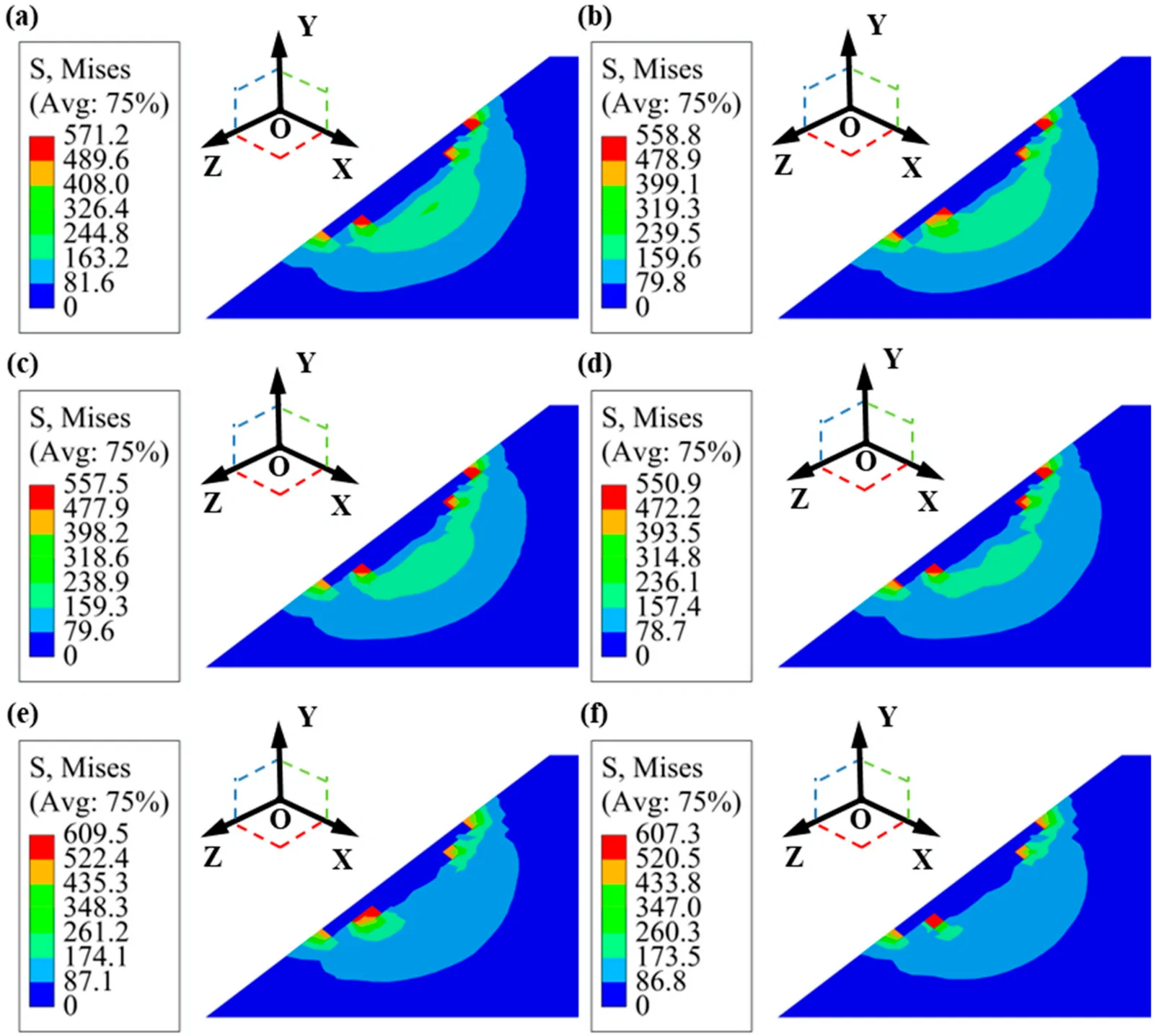
Von Mises stress distributions in the workpiece during laser-assisted milling of GH4096 under different laser spot radii: (**a**) 1.6 mm, (**b**) 1.8 mm, (**c**) 2.0 mm, (**d**) 2.2 mm, (**e**) 2.4 mm, and (**f**) 2.6 mm.

Figure 14 presents the machined-surface von Mises equivalent stress distributions at the spot radii. The maximum surface von Mises equivalent stress, denoted by *σ*_s,max_, is 571.18, 558.77, 557.54, 550.89, 609.48, and 607.26 MPa at 1.6, 1.8, 2.0, 2.2, 2.4, and 2.6 mm, respectively. From 1.6 to 2.2 mm, *σ*_s,max_ decreases by 20.29 MPa, or 3.55%. When the radius increases from 2.2 to 2.4 mm, it rises by 58.59 MPa, or 10.64%, reaching the series maximum of 609.48 MPa. From 2.4 to 2.6 mm, the value decreases slightly by 2.22 MPa, or 0.36%, and remains at the high level of 607.26 MPa. Across the complete radius range, the endpoint value increases by 36.08 MPa, corresponding to 6.32% relative to the 1.6 mm condition, while the full series spans 58.59 MPa between its minimum and maximum values.

The spatial distributions show a corresponding staged evolution. The intermediate-radius cases exhibit morphologies close to the baseline condition. At 2.4 and 2.6 mm, the green surface region becomes smaller and the blue area becomes more extensive, while the local red–orange peaks become brighter and more concentrated. The 2.4 mm condition combines this concentrated high-stress morphology with the series maximum of 609.48 MPa, and the 2.6 mm condition retains a similar concentration pattern with a peak of 607.26 MPa. The equivalent stress response therefore forms a gradual decrease over 1.6–2.2 mm, a sharp increase at 2.4 mm, and a high-level continuation at 2.6 mm.

The three response families together characterize the effect of spot radius. The *F*_90_ level decreases by 27.63% from 1.6 to 2.4 mm and rebounds by 8.06% at 2.6 mm. The endpoint final-frame regional mean and *T*_90_ decrease by 13.52% and 26.46%, respectively, while the common-region *T*_c_ increases by 1.10% between the two endpoint radii; these comparisons involve different spatial and temporal summaries. In contrast, *σ*_s,max_ reaches its maximum at 2.4 mm and remains nearly unchanged at 2.6 mm. This coordinated set of numerical and spatial features defines the radius-dependent force, temperature-field, and surface equivalent stress responses.

### 3.3. Cutting Depth

**Figure 15:**
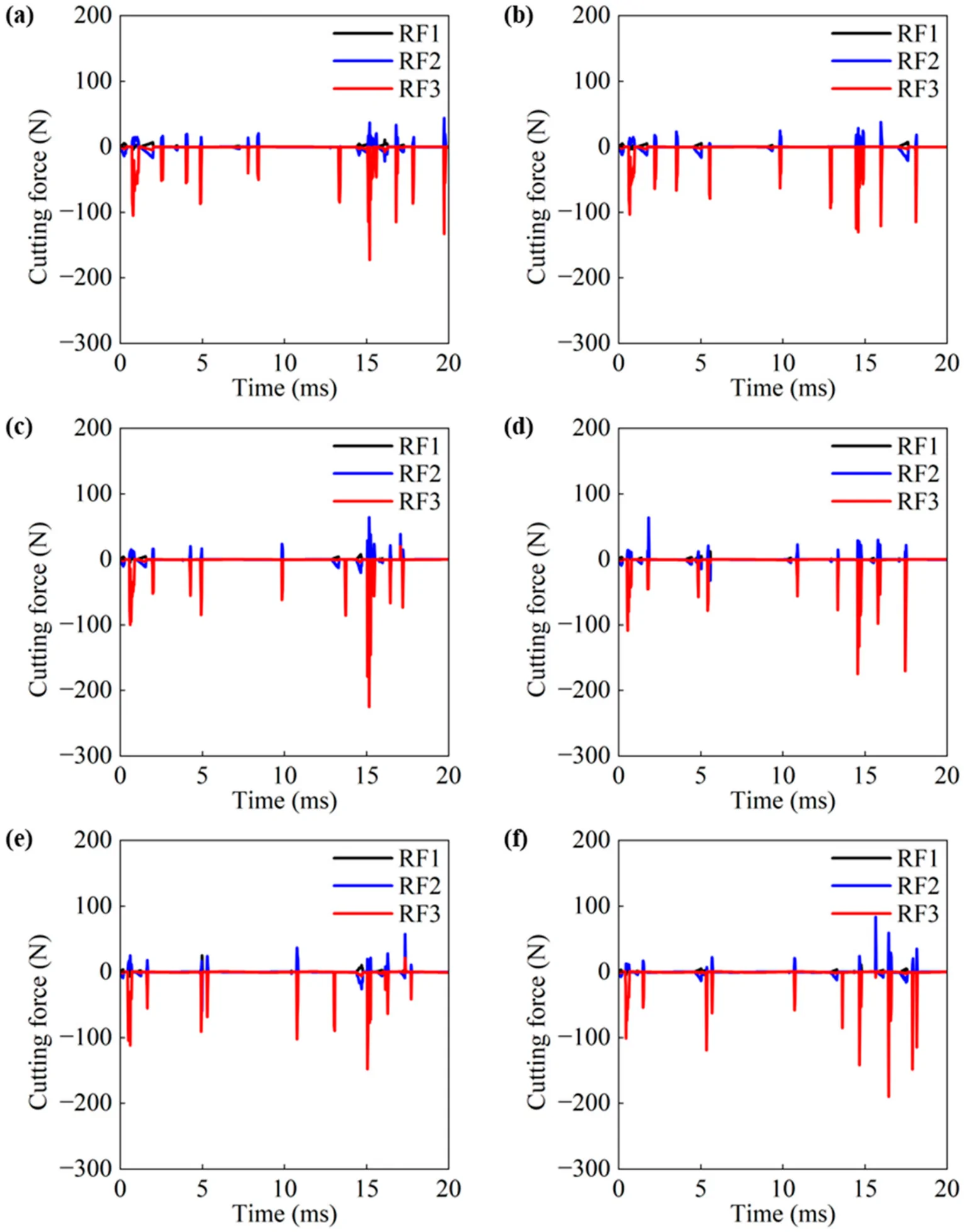
Time histories of the three milling-force components (*RF1*, *RF2*, and *RF3*) during laser-assisted milling of GH4096 under different cutting depths: (**a**) 0.4 mm, (**b**) 0.5 mm, (**c**) 0.6 mm, (**d**) 0.7 mm, (**e**) 0.8 mm, and (**f**) 0.9 mm.

Figure 15 presents the milling-force curves at cutting depths of 0.4, 0.5, 0.6, 0.7, 0.8 and 0.9 mm in panels (a)–(f), respectively. *RF3* remains the most pronounced negative impact component in every case, *RF1* fluctuates only slightly around zero, and *RF2* appears mainly as positive pulses whose local peaks become more evident at the larger depths. Both the timing and magnitude of the impulses change appreciably with cutting depth. Using the representative strong-peak metric *F*_90_ defined in Section 2.5, *F*_90_ decreases from 58.64 N at 0.4 mm to 25.75 N at 0.8 mm, a reduction of 32.89 N or 56.09%. It then rises by 24.10 N or 93.59% to 49.85 N at 0.9 mm. The 0.9 mm value remains 8.79 N or 14.99% below the 0.4 mm value. Thus, the *F*_90_ sequence follows a pronounced decrease–rebound pattern, with the minimum at 0.8 mm and the rebound concentrated between 0.8 and 0.9 mm. At the same time, cutting depth changes the active ball-end engagement geometry, contact length and removed volume, so the force response varies jointly with the mechanical engagement state and the temperature response described below.

**Figure 16:**
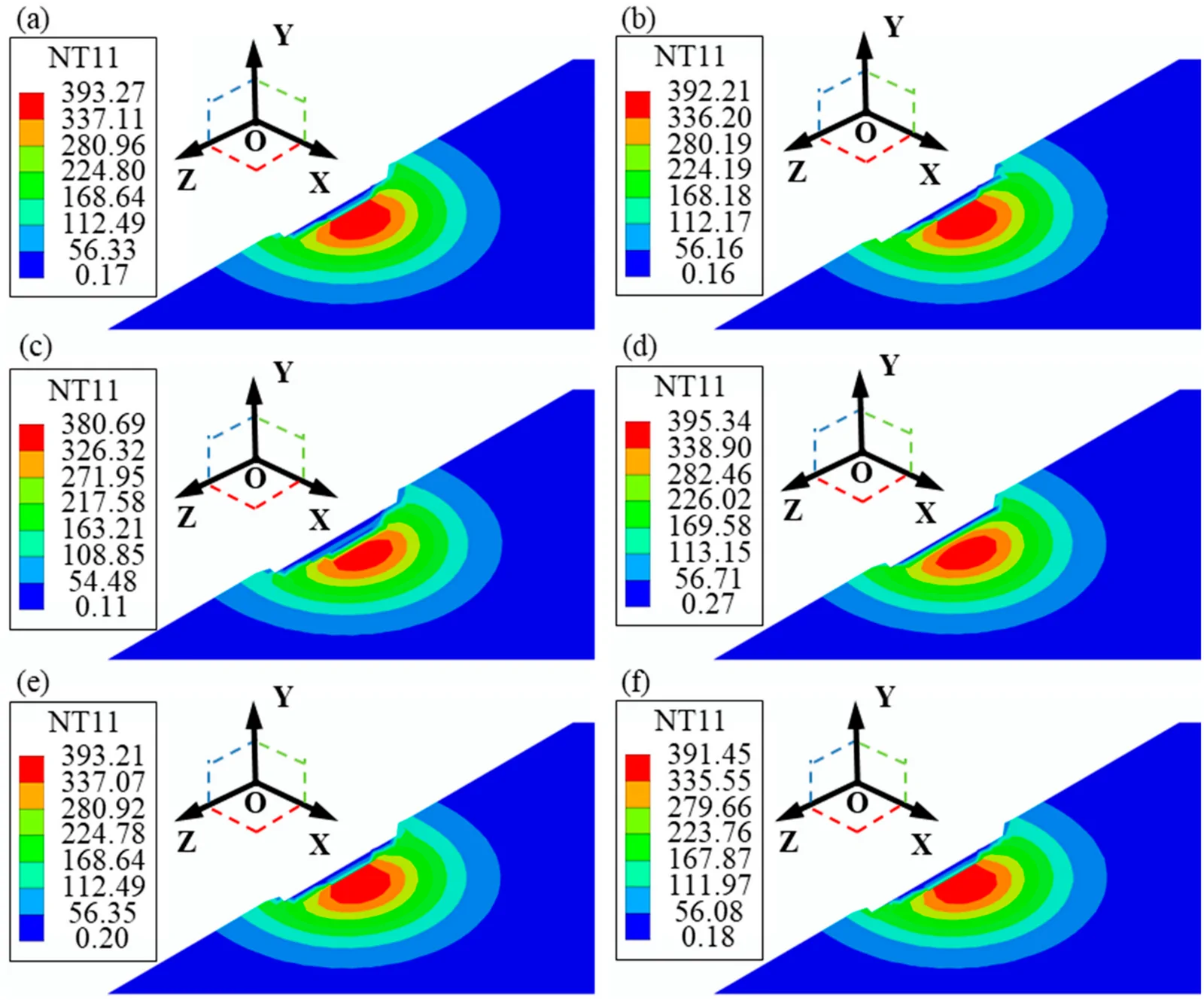
Temperature distributions during laser-assisted milling of GH4096 under different cutting depths: (**a**) 0.4 mm, (**b**) 0.5 mm, (**c**) 0.6 mm, (**d**) 0.7 mm, (**e**) 0.8 mm, and (**f**) 0.9 mm.

Figure 16 shows the temperature distributions during laser-assisted milling of GH4096 at cutting depths ranging from 0.4 to 0.9 mm. The laser power, spot radius and feed speed remain fixed while cutting depth changes from 0.4 to 0.9 mm. The peak temperature *T*_p_ is 723.79, 566.83, 549.57, 637.41, 619.08 and 582.26 °C, respectively. *T*_p_ decreases by 156.96 °C or 21.69% from 0.4 to 0.5 mm and by a further 17.26 °C or 3.05% from 0.5 to 0.6 mm, reaching the minimum of 549.57 °C at 0.6 mm. It then increases by 87.84 °C or 15.98% at 0.7 mm, before decreasing by 18.33 °C or 2.88% at 0.8 mm and by 36.82 °C or 5.95% at 0.9 mm. The endpoint value at 0.9 mm is 141.53 °C or 19.55% below the value at 0.4 mm, while the full-series range is 174.22 °C between 0.6 and 0.4 mm.

The mean temperature remains within 54.10–56.65 °C, giving a total spread of 2.55 °C or 4.71% relative to the lower endpoint. In the common 3103-node region, *T_c_* increases from 41.68 °C at 0.6 mm to 43.46 °C at 0.7 mm, an increase of 1.78 °C or 4.27%; it then decreases to 42.78 °C at 0.9 mm, a decrease of 0.68 °C or 1.56% relative to 0.7 mm. The temperature indicators therefore form a local minimum–maximum-decrease sequence across the depth series: *T*_p_ is highest at 0.4 mm and lowest at 0.6 mm, whereas *T*_c_ is lowest at 0.6 mm and highest at 0.7 mm. These numerical changes describe the depth-dependent thermal response together with the engagement-geometry changes reflected in *F*_90_.

**Figure 17:**
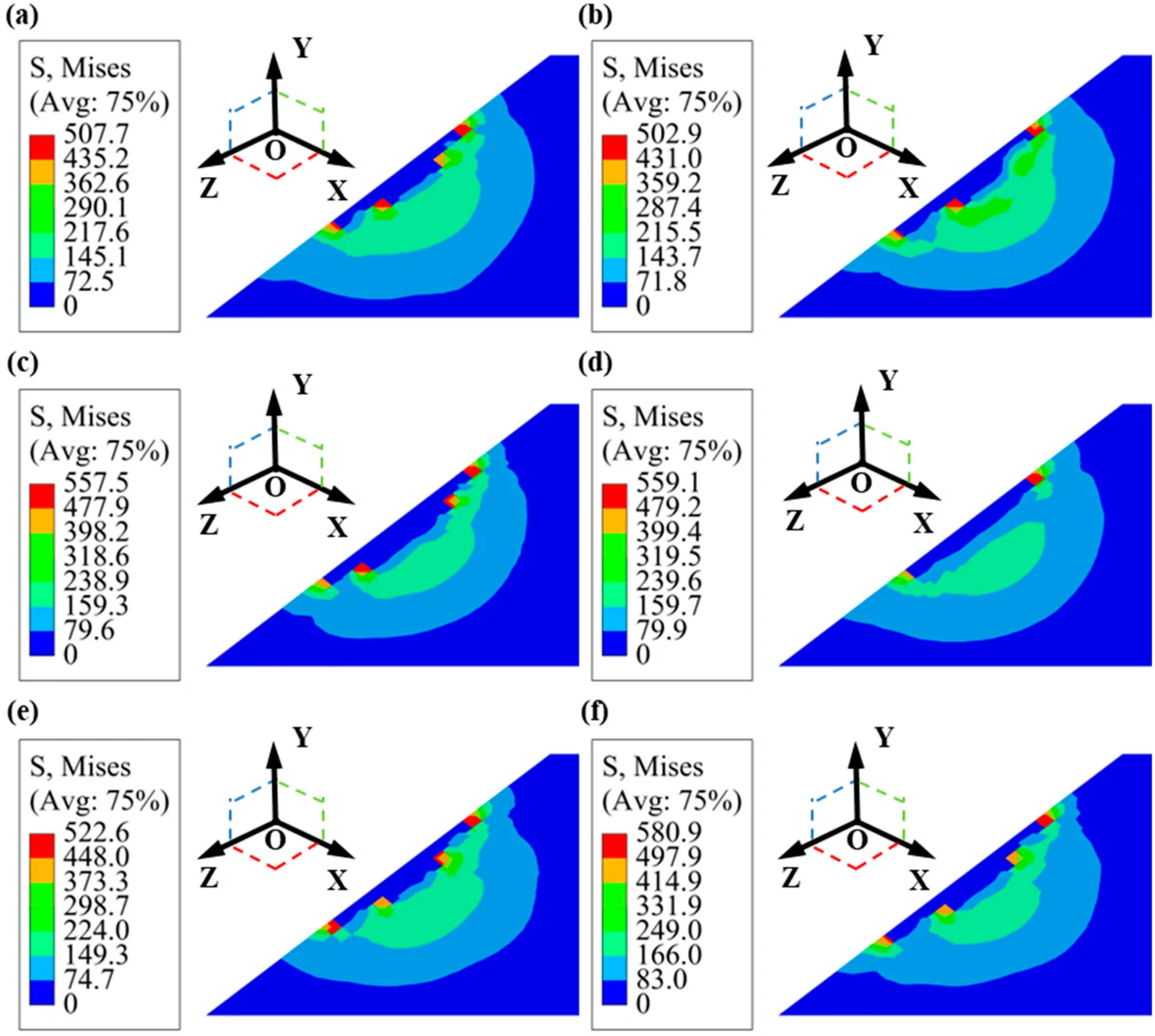
Von Mises stress distributions in the workpiece during laser-assisted milling of GH4096 under different cutting depths: (**a**) 0.4 mm, (**b**) 0.5 mm, (**c**) 0.6 mm, (**d**) 0.7 mm, (**e**) 0.8 mm, and (**f**) 0.9 mm.

Figure 17 shows the machined-surface von Mises equivalent stress distributions at cutting depths from 0.4 to 0.9 mm, with panels (a)–(f) corresponding to the same sequence. The maximum surface von Mises equivalent stress, denoted by *σ*_s,max_, is 507.69, 502.88, 557.54, 559.09, 522.64 and 580.90 MPa, respectively. From 0.4 to 0.5 mm, *σ*_s,max_ decreases by 4.81 MPa or 0.95%, reaching the series minimum of 502.88 MPa. It then increases by 54.66 MPa or 10.87% from 0.5 to 0.6 mm and by 1.55 MPa or 0.28% from 0.6 to 0.7 mm, reaching 559.09 MPa. From 0.7 to 0.8 mm, the value decreases by 36.45 MPa or 6.52% to 522.64 MPa, followed by an increase of 58.26 MPa or 11.15% at 0.9 mm, where the series maximum of 580.90 MPa occurs. Across the full 0.4–0.9 mm range, the endpoint increase is 73.21 MPa or 14.42%, and the difference between the series maximum and minimum is 78.02 MPa or 15.51% relative to the minimum.

The spatial distributions follow the same staged variation. At 0.4 and 0.5 mm, the arcuate subsurface affected layer is relatively thick and the green surface coverage is broad, while the local stress peaks remain weak. Increasing the depth to 0.6 and 0.7 mm is accompanied by more red–orange surface peaks and an increase in *σ*_s,max_ to 557.54 and 559.09 MPa. At 0.8 mm, the maximum decreases to 522.64 MPa. At 0.9 mm, red–orange peaks become the most pronounced and *σ*_s,max_ reaches 580.90 MPa. The force, temperature and stress sequences therefore exhibit distinct depth-dependent patterns: *F*_90_ reaches its minimum at 0.8 mm and rebounds at 0.9 mm, *T*_p_ reaches its minimum at 0.6 mm, *T*_c_ reaches its maximum at 0.7 mm, and *σ*_s,max_ reaches its maximum at 0.9 mm. Together, these numerical and spatial changes characterize the coupled response of the milling force, temperature field and machined-surface von Mises equivalent stress field to cutting depth.

### 3.4. Feed Speed

**Figure 18:**
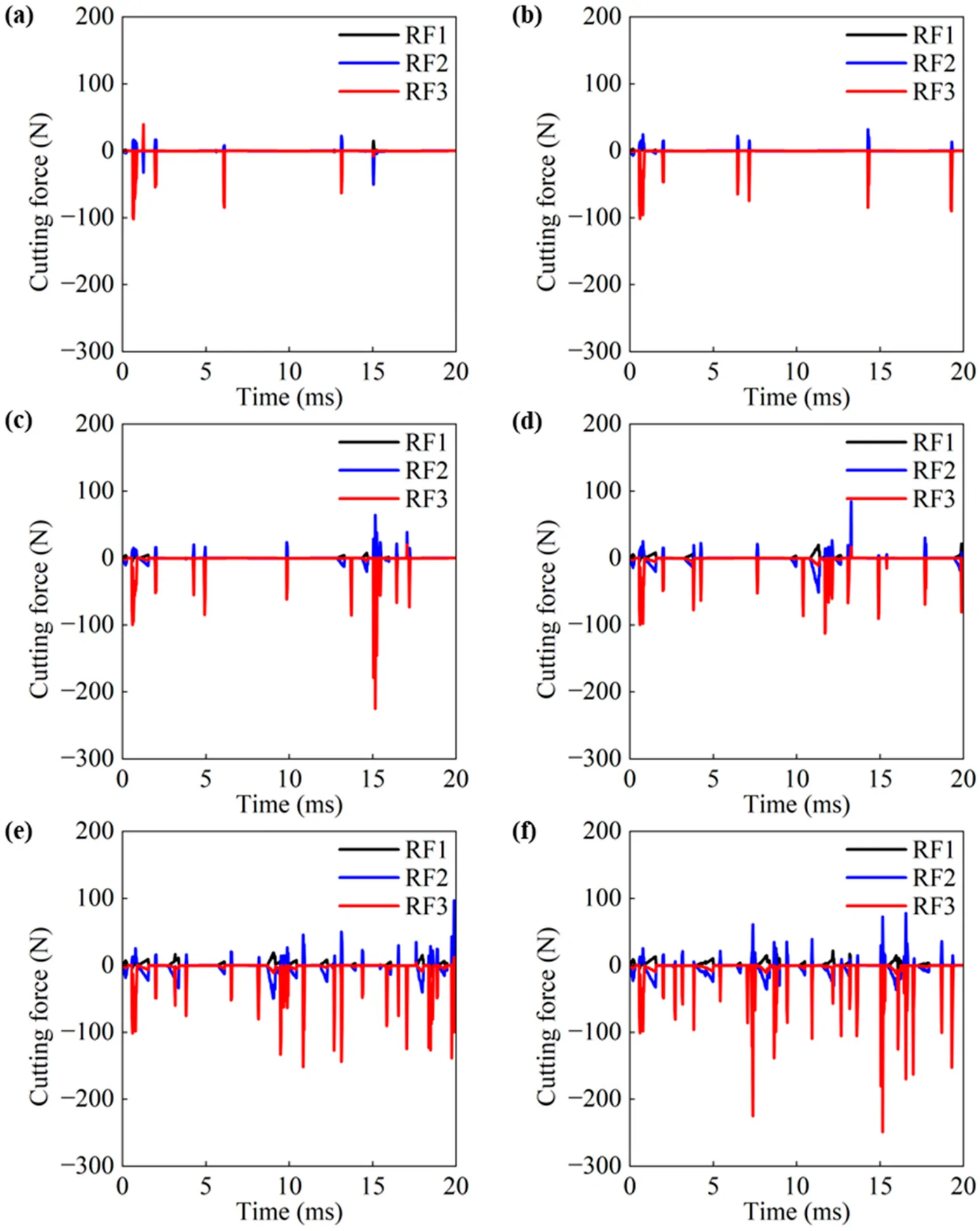
Time histories of the three milling-force components (*RF1*, *RF2*, and *RF3*) during laser-assisted milling of GH4096 under different feed speeds: (**a**) 5 mm/s, (**b**) 10 mm/s, (**c**) 15 mm/s, (**d**) 20 mm/s, (**e**) 25 mm/s, and (**f**) 30 mm/s.

Figure 18 presents the milling-force curves at feed speeds of 5, 10, 15, 20, 25, and 30 mm/s in panels (a)–(f), respectively. At low feed speeds, the three RF components exhibit relatively sparse impulses. As feed speed increases, the *RF1* and *RF2* fluctuations become denser, and the negative *RF3* impacts occur more frequently. The 25–30 mm/s conditions show the most continuous pulse activity across the feed-speed series.

*F*_90_ increases from 16.64 N at 5 mm/s to 51.06 N at 10 mm/s, a rise of 34.42 N, or 206.85%. It subsequently remains at a comparatively high level over 10–30 mm/s and reaches 53.20 N at 30 mm/s. The difference between 10 and 30 mm/s is 2.14 N, or 4.19%, placing the principal force transition between 5 and 10 mm/s. Across the full 5–30 mm/s interval, the endpoint increase is 36.56 N, or 219.71% relative to the 5 mm/s value. Within the investigated one-factor matrix, feed speed gives the largest baseline-normalized *F*_90_ range, at 73.0%. The force response therefore combines a pronounced initial increase with sustained high-amplitude loading at higher feed speeds.

**Figure 19:**
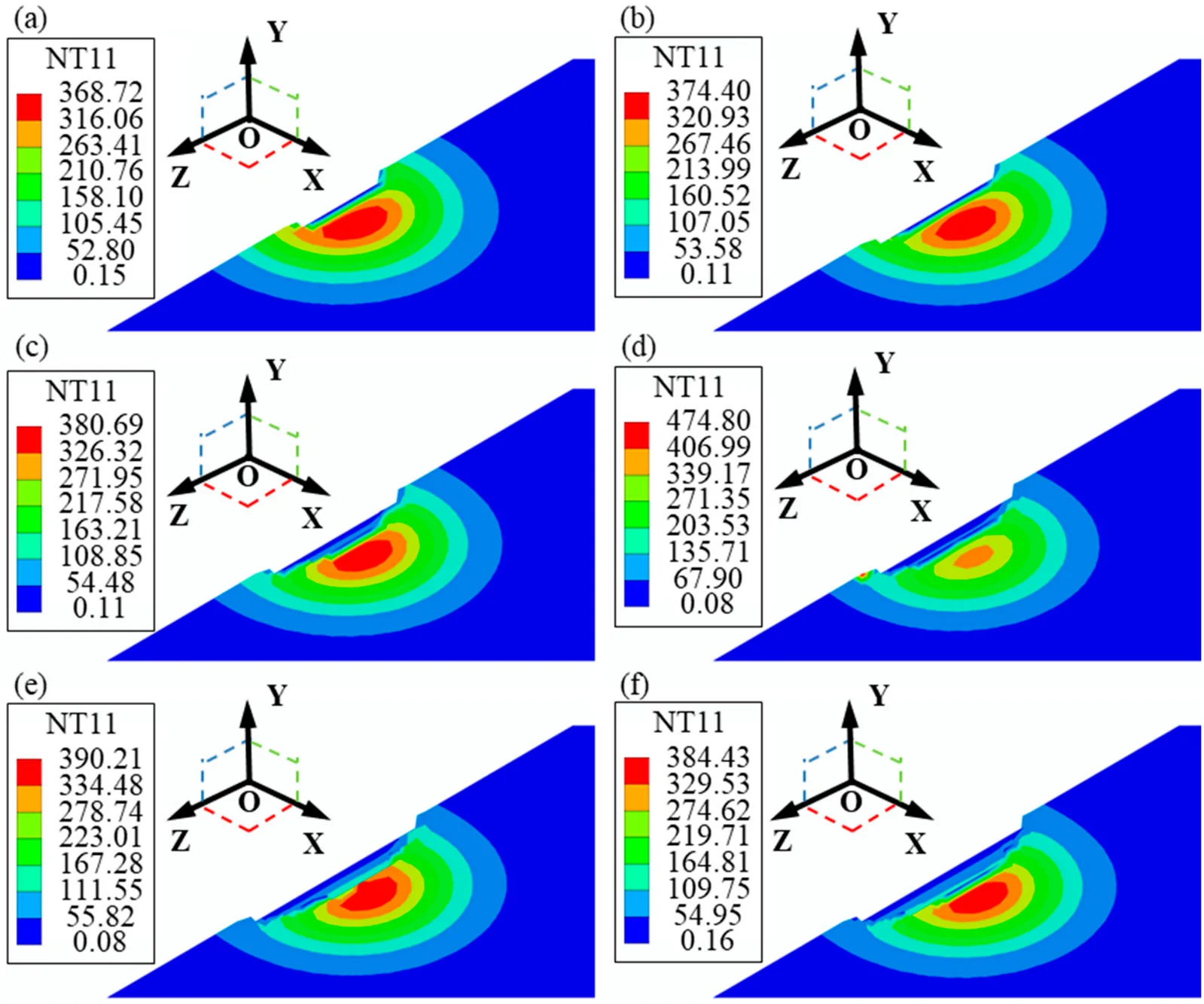
Temperature distributions during laser-assisted milling of GH4096 under different feed speeds: (**a**) 5 mm/s, (**b**) 10 mm/s, (**c**) 15 mm/s, (**d**) 20 mm/s, (**e**) 25 mm/s, and (**f**) 30 mm/s.

Figure 19 shows the temperature distributions during laser-assisted milling of GH4096 at feed speeds ranging from 5 to 30 mm/s. Feed speed produces contrasting changes in local peak temperature and regional heating. Over the full observation window, *T*_p_ increases from 432.88 °C at 5 mm/s to 1155.88 °C at 30 mm/s. This endpoint increase is 723.00 °C, corresponding to 167.02% relative to the 5 mm/s value. Across the same feed-speed interval, the final-frame regional mean decreases from 55.37 to 53.29 °C, a reduction of 2.08 °C, or 3.76%. In the common 3011-node region, *T*_c_, evaluated over the full time window, ranges from 42.10 to 42.52 °C, spanning 0.42 °C. Thus, the local peak increases markedly while the common-region temperature statistic remains within a narrow range. At *T*_p_ = 1155.88 °C, the thermal factor calculated from the model in Section 2.5 is *S*_T_ = 0.3098.

The travel-matched comparison further resolves the regional temperature response at a nominal travel of 0.1 mm. The corresponding observation times are 20.000 ms at 5 mm/s and 3.333 ms at 30 mm/s. At these respective times, the case-specific regional mean decreases from 55.37 to 32.59 °C, a reduction of 22.78 °C, or 41.14%. The *T*_90_ decreases from 156.76 to 69.71 °C, a reduction of 87.05 °C, or 55.53%. The upper-tail regional temperature therefore decreases more strongly than the regional mean over the same nominal travel. Together, the two comparisons distinguish an increasing full-window local peak from lower regional temperatures at matched travel as feed speed rises.

**Figure 20:**
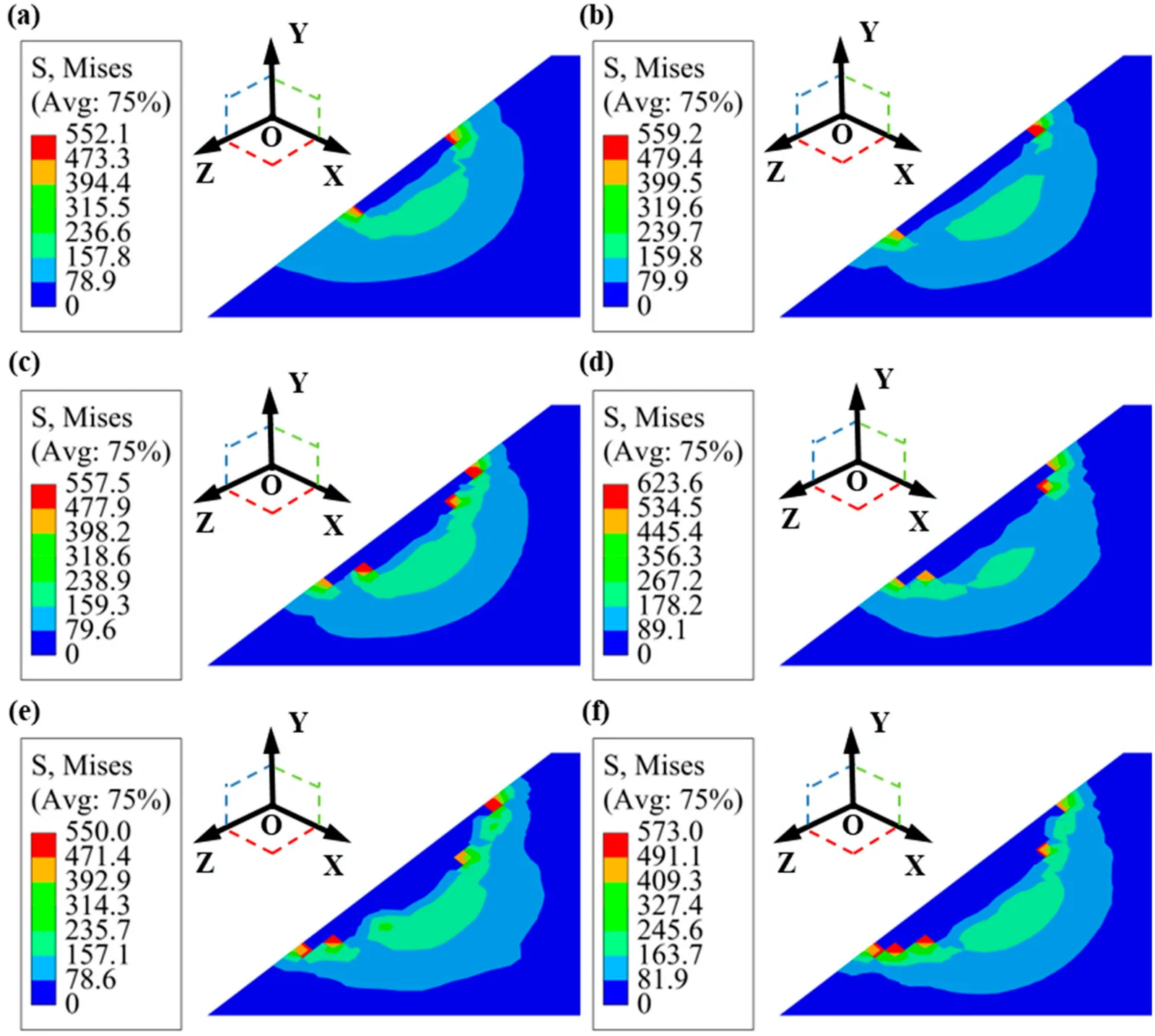
Von Mises stress distributions in the workpiece during laser-assisted milling of GH4096 under different feed speeds: (**a**) 5 mm/s, (**b**) 10 mm/s, (**c**) 15 mm/s, (**d**) 20 mm/s, (**e**) 25 mm/s, and (**f**) 30 mm/s.

Figure 20 presents the machined-surface von Mises equivalent stress distributions for the same feed-speed series as Figure 12. At 5, 10, 15, 20, 25, and 30 mm/s, *σ*_s,max_ is 552.14, 559.24, 557.54, 623.56, 549.99, and 529.32 MPa, respectively. Over 5–15 mm/s, the values occupy a narrow 552.14–559.24 MPa band, with a spread of 7.10 MPa. Increasing feed speed from 15 to 20 mm/s raises *σ*_s,max_ by 66.02 MPa, or 11.84%, to the series maximum of 623.56 MPa. From 20 to 25 mm/s, *σ*_s,max_ decreases by 73.57 MPa, or 11.80%, followed by a further decrease of 20.67 MPa, or 3.76%, at 30 mm/s. The total reduction from 20 to 30 mm/s is 94.24 MPa, or 15.11%. The 30 mm/s condition gives the series minimum, which is 22.82 MPa, or 4.13%, below the value at 5 mm/s. In the 30 mm/s panel, the 573.0 MPa color-bar limit represents the whole-model maximum, while the machined-surface maximum is 529.32 MPa.

The spatial distributions complement this narrow-band–peak–decline sequence. At 20 mm/s, the most prominent local high-stress zones coincide with the maximum *σ*_s,max_. At 25 mm/s, the green gradient band extends along the inclined surface, and the high-stress peaks become more dispersed. At 30 mm/s, several localized peaks remain visible alongside the lower surface maximum. The highest feed speed therefore combines a high *F*_90_ and elevated *T*_p_ with the lowest *σ*_s,max_ in the series. These paired trends distinguish the feed-speed dependence of cutting-stage force and temperature peaks from that of the maximum surface von Mises equivalent stress at the final recorded time.

### 3.5. Spindle Speed

**Figure 21:**
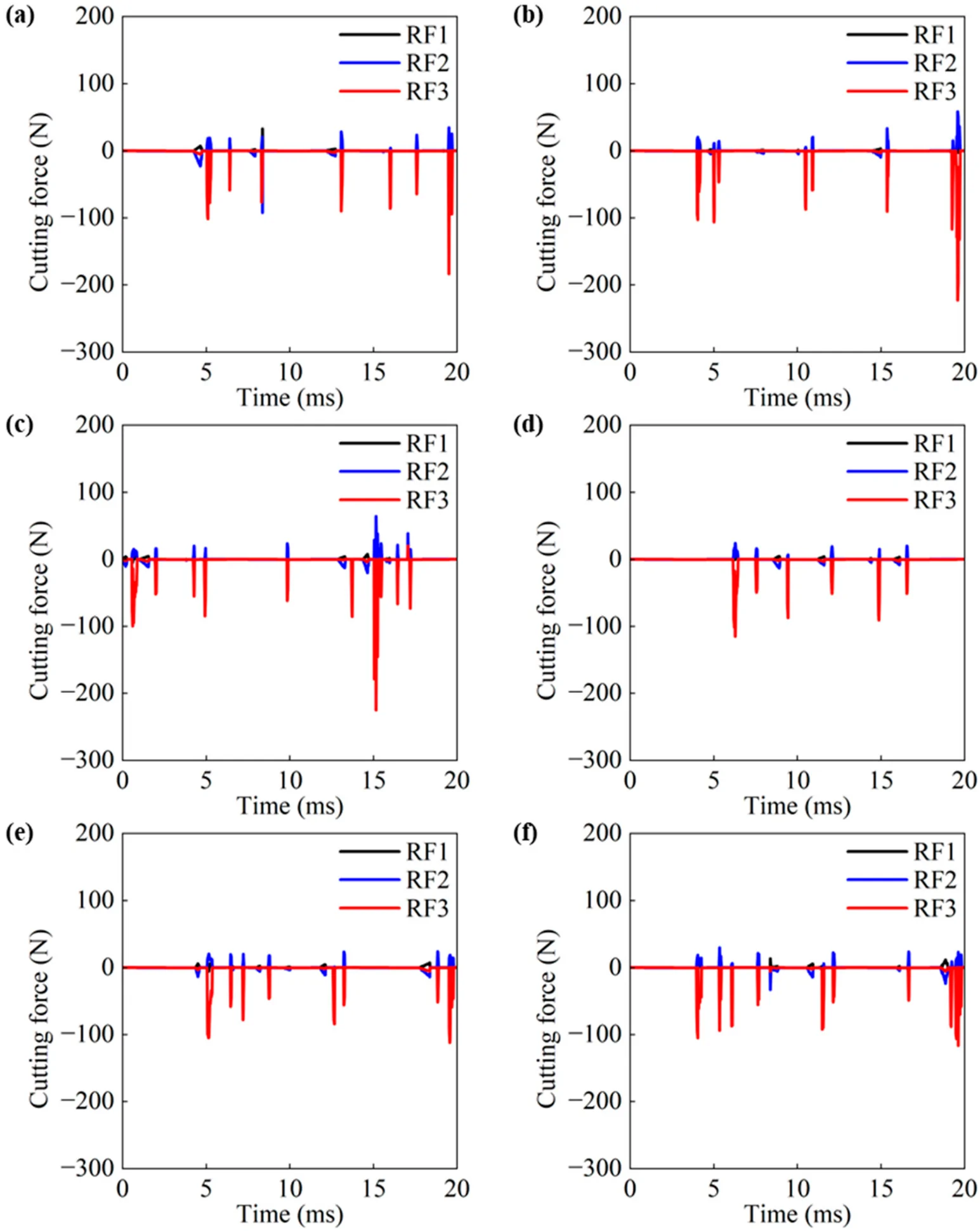
Time histories of the three milling-force components (*RF1*, *RF2*, and *RF3*) during laser-assisted milling of GH4096 under different spindle speeds: (**a**) 7000 r/min, (**b**) 8000 r/min, (**c**) 9000 r/min, (**d**) 10,000 r/min, (**e**) 11,000 r/min, and (**f**) 12,000 r/min.

Figure 21 presents the milling-force curves at spindle speeds of 7000, 8000, 9000, 10,000, 11,000, and 12,000 r/min in panels (a)–(f), respectively. *RF3* remains the dominant impact component with negative values, whereas *RF1* and *RF2* have smaller amplitudes throughout the speed series. The pulse spacing and density vary with spindle speed, reflecting changes in tooth-engagement frequency. *F*_90_ increases from 40.32 N at 7000 r/min to 50.11 N at 9000 r/min, an increase of 9.79 N (24.28%). It then decreases to 35.96 N at 12,000 r/min, a reduction of 14.15 N (28.24%) relative to the 9000 r/min value. The endpoint value is 4.36 N (10.81%) lower than that at 7000 r/min. Thus, *F*_90_ exhibits a rise–fall pattern, with its maximum at 9000 r/min. At fixed feed speed, increasing spindle speed reduces feed per tooth and increases cutting velocity.

**Figure 22:**
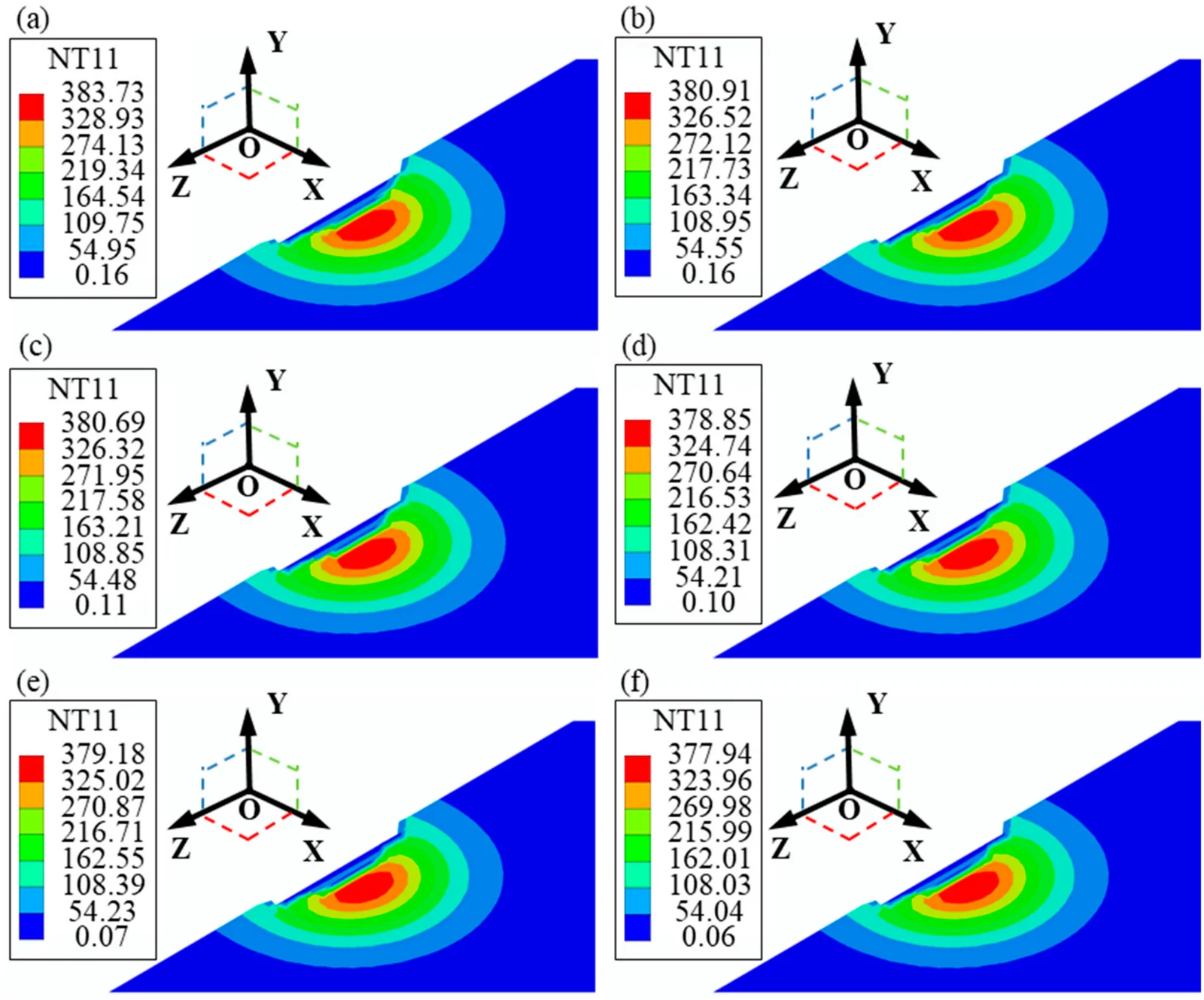
Temperature distributions during laser-assisted milling of GH4096 under different spindle speeds: (**a**) 7000 r/min, (**b**) 8000 r/min, (**c**) 9000 r/min, (**d**) 10,000 r/min, (**e**) 11,000 r/min, and (**f**) 12,000 r/min.

Figure 22 shows small endpoint changes alongside fluctuations across the spindle-speed series. From 7000 to 12,000 r/min, the mean temperature decreases from 54.28 to 53.87 °C, giving an endpoint difference of 0.41 °C. In the common 3100-node region, *T*_c_ changes from 41.91 to 41.55 °C, corresponding to a net decrease of 0.36 °C. Both regional temperature measures exhibit local reversals across the intermediate speeds. By contrast, *T*_p_ follows a non-monotonic sequence, with a minimum of 407.29 °C at 11,000 r/min and a maximum of 606.86 °C at 12,000 r/min. The difference between these two local-peak temperatures is 199.57 °C. The temperature response therefore combines small endpoint shifts in the regional measures with pronounced variation in the local peak. With laser power, radius, and feed speed fixed, the prescribed laser energy per unit travel remains constant throughout the speed series.

**Figure 23:**
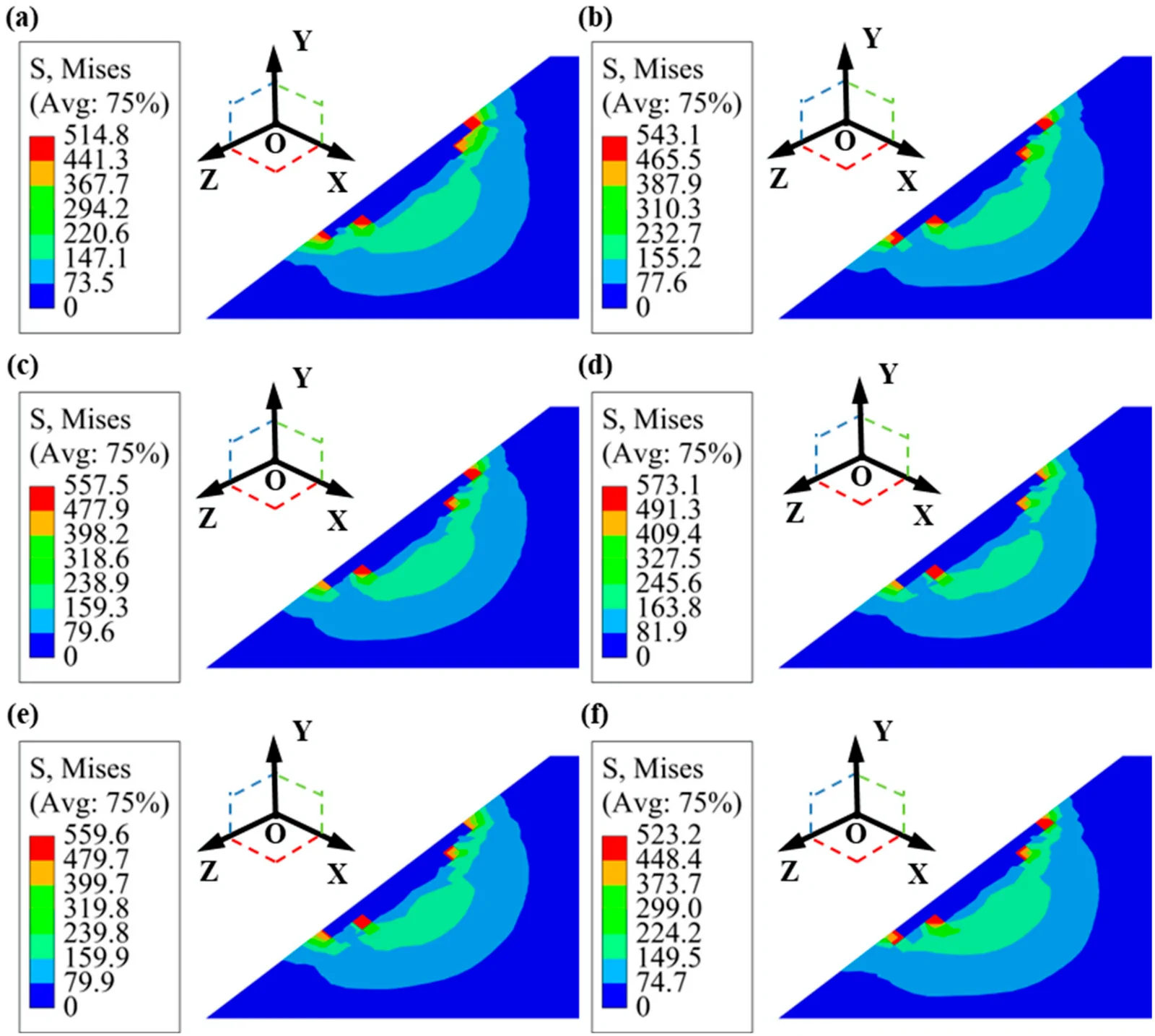
Von Mises stress distributions in the workpiece during laser-assisted milling of GH4096 under different spindle speeds: (**a**) 7000 r/min, (**b**) 8000 r/min, (**c**) 9000 r/min, (**d**) 10,000 r/min, (**e**) 11,000 r/min, and (**f**) 12,000 r/min.

Figure 23 presents the machined-surface von Mises equivalent stress distributions at the same spindle speeds as Figure 14. The maximum surface von Mises equivalent stress, *σ*_s,max_, is 514.79, 543.07, 557.54, 573.14, 559.60, and 523.19 MPa, respectively. From 7000 to 10,000 r/min, *σ*_s,max_ increases by 58.35 MPa (11.33%), reaching the series maximum of 573.14 MPa. It then decreases by 49.95 MPa (8.72%) to 523.19 MPa at 12,000 r/min. The final value remains 8.40 MPa (1.63%) above that at 7000 r/min. This rise–fall sequence places the maximum equivalent stress at 10,000 r/min, compared with 9000 r/min for *F*_90_.

The overall stress-band morphology remains similar across the six cases. Spatial differences appear mainly in the intensity of the local red–orange peaks and the extent of the green gradient zone. Together, the numerical values and spatial distributions characterize a spindle-speed response dominated by changes in peak stress and local gradients within a broadly similar banded pattern. Across the series, the maxima of *F*_90_, *σ*_s,max_, and *T*_p_ occur at 9000, 10,000, and 12,000 r/min, respectively. These distinct peak positions describe the different spindle-speed dependence of milling force, surface von Mises equivalent stress, and local temperature.

### 3.6. Coupled Effects of Laser Power and Spot Radius

To investigate the coupled effects of laser power and spot radius, six parameter combinations (S1–S6) are designed using two power levels and three spot radii, while keeping the other machining parameters constant (as Table 4 shows). The coupled influence of these two laser parameters is evaluated through their effects on milling force, temperature field, and surface von Mises equivalent stress.

**Figure 24:**
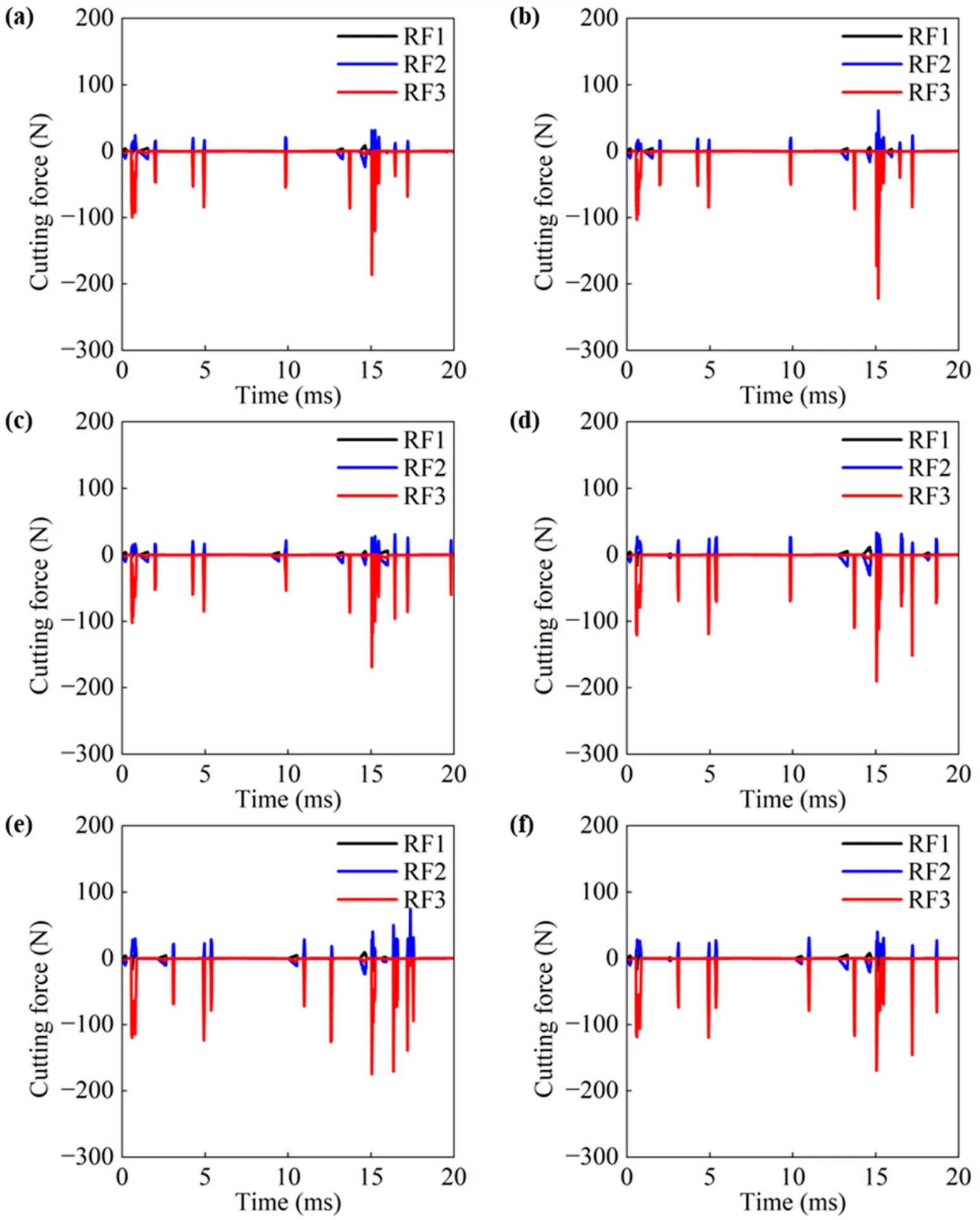
Time histories of the three milling-force components (RF1, RF2, and RF3) during laser-assisted milling of GH4096, illustrating the coupled effects of laser power and spot radius: (**a**) S1, (**b**) S2, (**c**) S3, (**d**) S4, (**e**) S5, and (**f**) S6.

Figure 24 presents the milling-force of S1–S6. At 250 W, the *F*_90_ follows 37.39 → 39.43 → 35.00 N as the laser spot radius increases from 1.6 to 2.2 and 2.4 mm; at 400 W, it increases from 42.24 to 64.19 and 69.48 N. Raising the laser power from 250 to 400 W changes the peak force by 12.97%, 62.79%, and 98.51% at the three radii, respectively. The effect of power therefore depends on spot radius, with a relatively small increase in *F*_90_ at 1.6 mm and substantially larger increases at 2.2 and 2.4 mm. A descriptive two-factor sum-of-squares decomposition of the six *F*_90_ values attributes 61.58%, 17.90%, and 20.52% of the total variation to laser power, spot radius, and their interaction, respectively. These results suggest that energy concentration and the fraction of heated elements within the cutting region jointly influence the balance between thermal softening and mechanical engagement.

**Figure 25:**
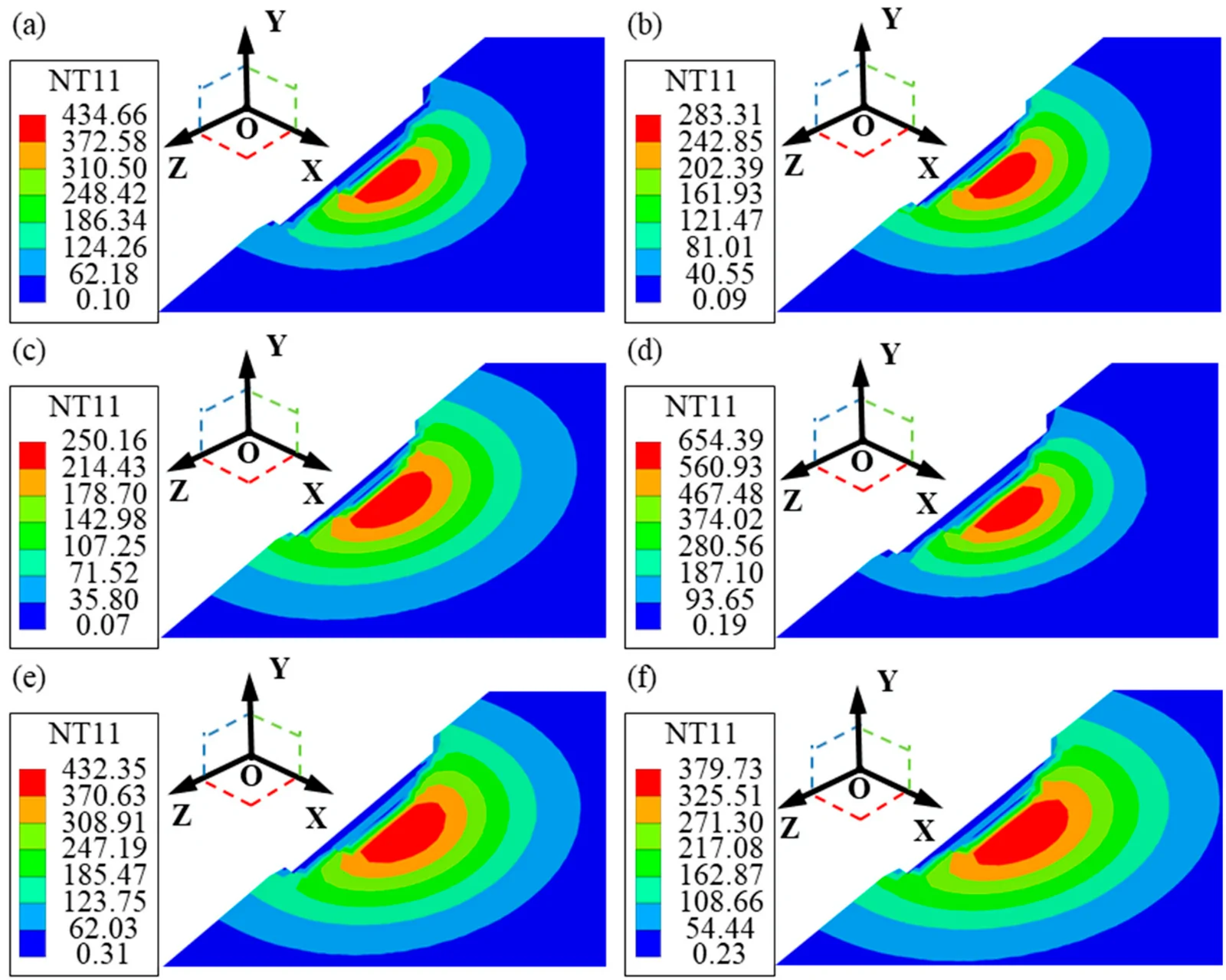
Temperature distributions during laser-assisted milling of GH4096, illustrating the coupled effects of laser power and spot radius: (**a**) S1, (**b**) S2, (**c**) S3, (**d**) S4, (**e**) S5, and (**f**) S6.

Increasing laser power primarily raises the common-region mean temperature, while the peak temperature is also sensitive to laser spot radius and its coupling with laser power. Changes in spot radius therefore affect localized temperature extremes more strongly than the regional mean. At 400 W, the peak temperature follows 791.93 → 572.48 → 793.77 °C (as Figure 25 shows), and the *S*_T_ changes from 0.8266 to 0.9477 and 0.8251. This nonmonotonic response may reflect the combined effects of energy concentration and heating extent. A smaller spot concentrates the laser input, whereas a larger spot distributes it over a wider area, altering the spatial overlap between heating and material removal and thus the local thermal response.

**Figure 26:**
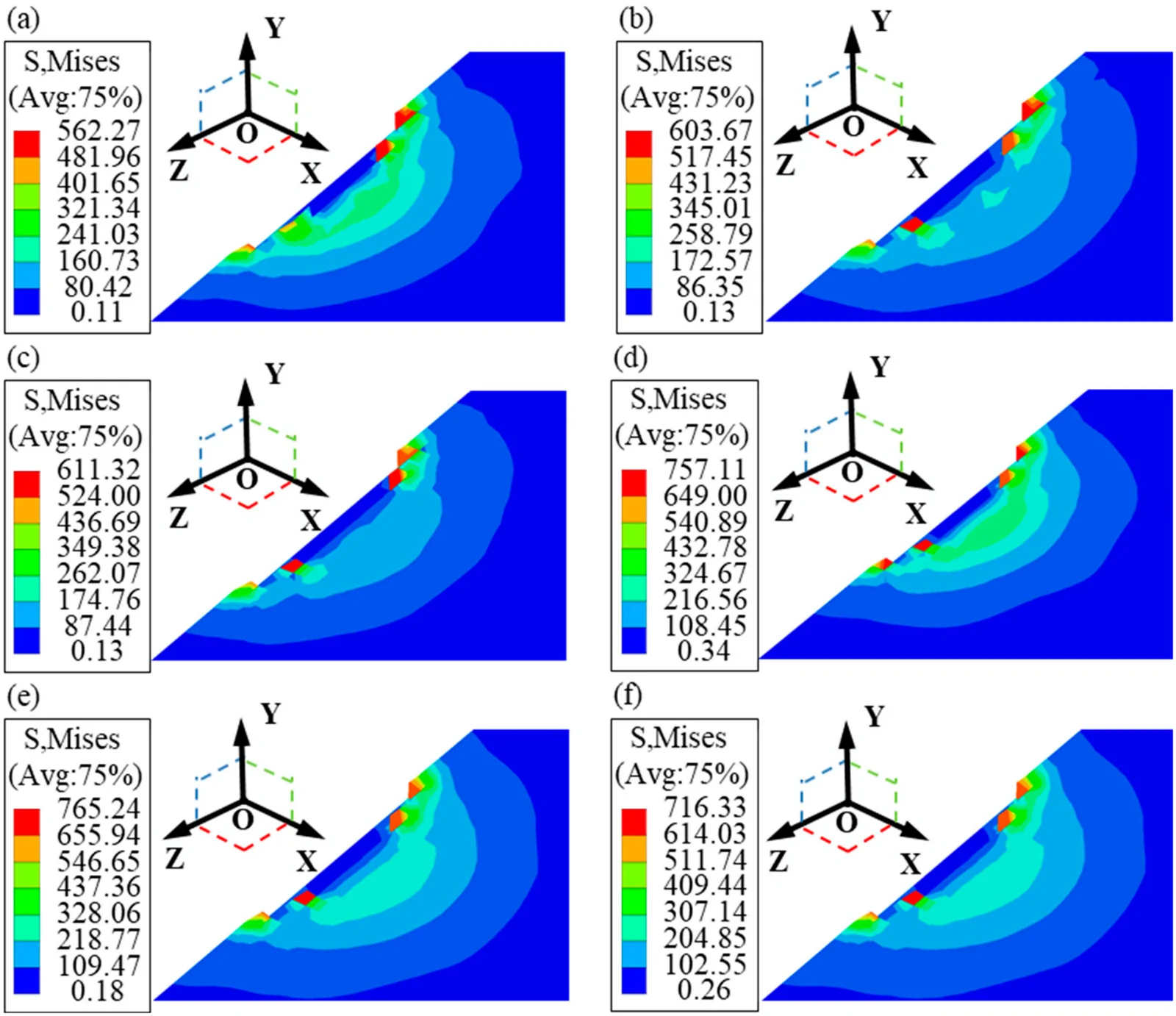
Von Mises stress distributions after laser-assisted milling of GH4096, illustrating the coupled effects of laser power and spot radius: (**a**) S1, (**b**) S2, (**c**) S3, (**d**) S4, (**e**) S5, and (**f**) S6.

Across the three spot radii, increasing laser power from 250 to 400 W raises the maximum final-frame surface von Mises equivalent stress from 562.27–611.32 MPa to 716.33–765.24 MPa, with respective increases of 34.65%, 26.76%, and 17.18%. At 400 W, S5 exhibits an *S*_T_ of 0.9477, an *F*_90_ of 64.19 N, and a maximum stress of 765.24 MPa. S6 reaches a softening index of 0.8251 and an *F*_90_ of 69.48 N, while its maximum stress decreases by 6.39% relative to S5(as Figure 26 shows). These results suggest that laser energy concentration and heating extent influence the temperature distribution and thermal softening, while milling force characterizes mechanical loading. Their combined effects on plastic deformation and subsequent thermal contraction may explain the nonmonotonic stress variations across S1–S6.

## 4. Conclusions

This work develops a three-dimensional thermomechanically coupled finite-element model for laser-field-assisted hybrid milling of powder-metallurgy nickel-based superalloy GH4096 for high-performance aero-engine components. The model incorporates a GH4096-specific material description calibrated using quasi-static, high-strain-rate, elevated-temperature and stress-state-dependent data, integrating JC plasticity, JC damage initiation, displacement-based fracture evolution and a moving Gaussian surface heat source. It simulates localized laser energy input, transient heat transfer, intermittent mechanical milling, plastic and frictional heating, and material separation to predict milling forces and surface von Mises equivalent stresses. The effects of laser power, spot radius, cutting depth, feed speed and spindle speed, together with the coupling between laser parameters, are investigated. The following conclusions can be drawn:

(1) Feed speed produces the largest peak-force variation relative to the baseline case (73.0%) within 5–30 mm/s. The representative strong-peak level rises from 16.64 N at 5 mm/s to 51.06 N at 10 mm/s, reaching 53.20 N at 30 mm/s; pulses are most continuous at 25–30 mm/s. Peak temperature rises from 432.88 to 1155.88 °C, while the common-region mean remains within 42.10–42.52 °C; the mean temperature decreases from 55.37 to 32.59 °C. Maximum surface von Mises equivalent stress is 552.14, 559.24, 557.54, 623.56, 549.99, and 529.32 MPa, peaking at 20 mm/s before falling by 94.24 MPa. Local stress peaks are strongest at 20 mm/s, become dispersed along the extended intermediate-stress band at 25 mm/s, and remain isolated at 30 mm/s.

(2) Cutting depth generates the second-largest peak-force variation relative to the baseline case (65.6%) within 0.4–0.9 mm. The representative strong-peak level changes from 58.64 N at 0.4 mm to 25.75 N at 0.8 mm and then to 49.85 N at 0.9 mm. Peak temperature varies non-monotonically, reaching 723.79 °C at 0.4 mm and its minimum of 549.57 °C at 0.6 mm, while the final-frame regional mean remains within 54.10–56.65 °C. Maximum surface von Mises equivalent stress is 507.69, 502.88, 557.54, 559.09, 522.64, and 580.90 MPa, with the minimum at 0.5 mm and maximum at 0.9 mm. The 0.4–0.5 mm cases show broad intermediate-stress coverage and weak local peaks; larger depths produce more red–orange peaks, with the strongest localization at 0.9 mm.

(3) Laser power produces a 29.7% peak-force variation relative to the baseline case over 200–450 W. The representative strong-peak level decreases from 50.11 N at 300 W to 35.20 N at 400 W, then increases to 47.23 N at 450 W. Peak temperature increases continuously from 498.12 to 647.21 °C, accompanied by a common-region mean increase from 38.17 to 53.24 °C and a *p*_50_ increase from 7.25% to 15.72%. Maximum surface von Mises equivalent stress is 599.45, 545.33, 557.54, 599.39, 619.75, and 559.88 MPa, first decreasing, then reaching its maximum at 400 W before falling by 59.87 MPa. The stress band is narrow at 200 W, widens at 350–400 W, and remains broad at 450 W despite the lower maximum.

(4) Laser spot radius produces a 28.8% peak-force variation relative to the baseline case over 1.6–2.6 mm. The representative strong-peak level decreases from 52.27 N at 1.6 mm to 37.83 N at 2.4 mm, then increases to 40.88 N at 2.6 mm. The final-frame regional mean and *T*_90_ decrease from 57.39 to 49.63 °C and from 166.41 to 122.37 °C, respectively, while the common-region mean changes only slightly. Maximum surface von Mises equivalent stress decreases from 571.18 MPa at 1.6 mm to 550.89 MPa at 2.2 mm, then rises sharply to 609.48 and 607.26 MPa at 2.4 and 2.6 mm. Within the present coupled model, these larger radii produce smaller intermediate-stress regions but more concentrated local high-stress peaks.

(5) Spindle speed produces a 28.2% peak-force variation relative to the baseline case over 7000–12,000 r/min. The representative strong-peak level rises from 40.32 N at 7000 r/min to 50.11 N at 9000 r/min, then falls to 35.96 N at 12,000 r/min. Peak temperature ranges from 407.29 °C at 11,000 r/min to 606.86 °C at 12,000 r/min, whereas the final-frame regional and common-region means show endpoint decreases of only 0.41 and 0.36 °C. Maximum surface von Mises equivalent stress rises from 514.79 MPa to 573.14 MPa at 10,000 r/min, then decreases to 523.19 MPa. The overall stress-band shape remains similar, with changes mainly in local peak intensity and intermediate-stress coverage.

(6) Laser power and spot radius exhibit coupled effects across S1–S6. A descriptive sum-of-squares decomposition attributes 61.58%, 17.90%, and 20.52% of the *F*_90_ variation to laser power, spot radius, and their interaction, respectively. Raising power from 250 to 400 W increases maximum surface von Mises equivalent stress by 34.65%, 26.76%, and 17.18% at the three radii. At 400 W, increasing the spot radius from 2.2 to 2.4 mm reduces the *S*_T_ from 0.9477 to 0.8251 and increases the fraction of heated elements within the cutting region from 64.48% to 70.35%, while increasing *F*_90_ from 64.19 to 69.48 N by 8.24% and reducing the maximum equivalent stress by 6.39% from 765.24 MPa. These responses reflect the combined effects of energy concentration, fraction of heated elements within the cutting region, and mechanical loading.

## Figures and Tables

**Table 1:** Constitutive-parameter comparison with existing references [17,26]. *A* and *B* are in MPa; *n*, *C* and *m* are dimensionless. The FGH96 row belongs to a modified JC formulation and is not interchangeable with the present constant-m JC model.

Material and Source	*A*	*B*	*n*	*C*	*m*
GH4096, present study	907.95	1421.96	0.5	0.017	3.56
Inconel 718, standard JC set [23]	450	1700	0.65	0.017	0.17
FGH96, modified JC set [26]	773	1271	0.667	0.031	8.05 × 10^−5^ $\dot{\varepsilon }$ + 1.66

**Table 2:** Johnson–Cook damage-parameter comparison with an existing Inconel 718 reference [28]. *D*_1_–*D*_5_ are dimensionless.

Material and Source	*D*_1_	*D*_2_	*D*_3_	*D*_4_	*D*_5_
GH4096, present study	−0.18	0.44	−0.41	0.04	0.63
Inconel 718 [28]	0.239	0.456	−0.3	0.07	2.5

**Table 3:** Selected process parameters and corresponding levels for laser-assisted milling of GH4096 nickel-based superalloy.

Parameter Class	Level 1	Level 2	Level 3	Level 4	Level 5	Level 6
Laser power (W)	200	250	300	350	400	450
Spot radius (mm)	1.6	1.8	2.0	2.2	2.4	2.6
Cutting depth (mm)	0.4	0.5	0.6	0.7	0.8	0.9
Feed speed (mm/s)	5	10	15	20	25	30
Spindle speed (r/min)	7000	8000	9000	10,000	11,000	12,000

**Table 4:** Process parameter combinations (S1–S6) for investigating the coupled effects of laser power and laser spot radius with constant mechanical cutting parameters during laser-assisted milling of GH4096 nickel-based superalloy.

Parameter Class	S1	S2	S3	S4	S5	S6
Laser power (W)	250	250	250	400	400	400
Spot radius (mm)	1.6	2.2	2.4	1.6	2.2	2.4
Cutting depth (mm)	0.6	0.6	0.6	0.6	0.6	0.6
Feed speed (mm/s)	15	15	15	15	15	15
Spindle speed (r/min)	9000	9000	9000	9000	9000	9000

## Data Availability

The original contributions presented in this study are included in the article. Further inquiries can be directed to the corresponding author.

## Nomenclature

Abbreviations		
DIC	Digital image correlation	
HIF	Isothermal forging	
HIP	Hot isostatic pressing	
JC	Johnson–Cook	
LAM	Laser-assisted milling	
LVDT	Linear variable differential transformer	
SHPB	Split Hopkinson pressure bar	
SHTB	Split Hopkinson tension bar	
Symbols		
*A*, *B*, *n*	Johnson–Cook strain-hardening parameters	
*C*	Johnson–Cook strain-rate sensitivity coefficient	
*m*	Johnson–Cook constant thermal-softening exponent	
*D*_1_–*D*_5_	Johnson–Cook damage parameters	
*RF1*	Cutting force in the feed direction	
*RF2*	Cutting force in the radial direction	
*RF3*	Cutting force in the axial direction	
*T*_0_, *T*_m_	Reference and melting/normalization temperatures	
${\dot{\varepsilon }}_{0}$	Reference equivalent plastic strain rate	
*η*	Stress triaxiality, defined as mean stress divided by von Mises equivalent stress	
*η*_abs_	Laser absorptivity of the workpiece surface	
*F*_90_	90th percentile of pooled absolute peaks from the three milling-force components	
𝒫	Pooled set of absolute directional force peaks	
*Q*_0.90_	90th-percentile operator	
*T*_c_	Mean temperature of valid space–time samples in the common region	
*N*	Total number of valid space–time temperature samples in the common region	
*σ*_vM_	von Mises equivalent stress	

## References

[1] Lei H., Yin Z., Zhao L., Cheng J., Chen M., Liu Q., Yang D., Chen G., Hu J., Chen J. (2025). Repair-Mechanism and Strategy-Customization during Micro-Milling of Flawed Potassium Dihydrogen Phosphate. Int. J. Mech. Sci..

[2] Li F., Bai Y., Meng L., Fu R., Zhong Y., Du J., Yin F., Feng D. (2020). Impact of Aging Heat Treatment on Microstructure and Mechanical Properties of a Newly Developed GH4096 Disk Superalloy. Mater. Charact..

[3] Li F., Fu R., Bai Y., Meng L., Tan H., Zhong Y., Tian W., Du J., Tian Z. (2023). Effects of Initial Grain Size and Strengthening Phase on Thermal Deformation and Recrystallization Behavior of GH4096 Superalloy. Acta Metall. Sin..

[4] Lei H., Yin Z., He Z., Cheng J., Zhao L., Liu Q., Ding W., Yang D., Chen G., Chen M. (2025). Characterization-Method and Repair-Mechanism of Atomic Defects in Potassium Dihydrogen Phosphate. Int. J. Mech. Sci..

[5] Peng Z., Luo J., Zhao Y., Zhou L., Wang X., Zou J. (2025). Evolution of Microstructure during Static Recrystallisation in FGH96 Superalloy. Acta Metall. Sin..

[6] Liu Y., Wang M., Sun P., Yang G., Song W., Wang X. (2023). Effect of Solution Treatment on Microstructure Evolution of a Powder Metallurgy Nickel-Based Superalloy with Incomplete Dynamic Recrystallisation Microstructure. Metals.

[7] Wang H., Zhang J., Shang H., Sha A., Cheng Y., Duan H. (2023). Experiment and Modelling of the Pre-Strain Effect on the Creep Behaviour of P/M Ni-Based Superalloy FGH96. Materials.

[8] Wang X., Liu Q., Wang H., Li X., Wang Q., Song G., Wang M. (2026). Machining Behaviour and Milling Force Modelling of Powder Metallurgy Superalloy under Nanofluid Minimum Quantity Lubrication. Int. J. Adv. Manuf. Technol..

[9] Wang R., Yang D., Wang W., Wei F., Lu Y., Li Y. (2022). Tool Wear in Nickel-Based Superalloy Machining: An Overview. Processes.

[10] Lei H., Yan H., Cheng J., Zhao L., Yang D., Chen G., Hu J., Chen J., Chen M. (2026). Wear Characteristics and Mechanisms of CBN Ball-End Mills in Micro-Milling of Soft-Brittle KDP Crystals. J. Manuf. Process..

[11] Chaubey S.K., Gupta K. (2025). Review of EDM-Based Machining of Nickel–Titanium Shape Memory Alloys. Quantum Beam Sci..

[12] Painuly M., Singh R.P., Trehan R. (2025). Investigation into Electrochemical Machining of Aviation-Grade Inconel 625 Superalloy: An Experimental Study with Advanced Optimisation and Microstructural Analysis. Aircr. Eng. Aerosp. Technol..

[13] Cheng J., Lei H., Zhao L., Liu Q., Hu Y., Chen M. (2025). Balancing Surface Quality and Machining Efficiency in Recycling Process Achieved by Micro Ball-End Milling of Flawed Potassium Dihydrogen Phosphate Optics. Sustain. Mater. Technol..

[14] Gu G., Wang D., Wu S., Zhou S., Zhang B. (2024). Research Status and Prospect of Ultrasonic Vibration and Minimum Quantity Lubrication Processing of Nickel-Based Alloys. Intell. Sustain. Manuf..

[15] Kaynak Y., Lu T., Jawahir I.S. (2014). Cryogenic Machining-Induced Surface Integrity: A Review and Comparison with Dry, MQL, and Flood-Cooled Machining. Mach. Sci. Technol..

[16] Hu G., Lu Y., Zhou S., Zhang M., He X., Zhang F., Chen G. (2025). Experimental Study on Surface Integrity of Nickel-Based Superalloy in Ultrasonic Elliptical Vibration Cutting. Micromachines.

[17] Zhang H., Chen F., Li Z., Hu W., Sun T., Zhang J. (2023). Investigation of Laser-Assisted Micro-Milling Process of Inconel 718. J. Manuf. Mater. Process..

[18] Zhou S., Xu J., Zhao L., Yang Y., Li Z., Zhang J. (2025). Parametric Dependence of Thermal Field in Laser-Assisted Turning of GH4169. Optics.

[19] Zerilli F.J., Armstrong R.W. (1987). Dislocation-Mechanics-Based Constitutive Relations for Material Dynamics Calculations. J. Appl. Phys..

[20] Follansbee P.S., Kocks U.F. (1988). A Constitutive Description of the Deformation of Copper Based on the Use of the Mechanical Threshold Stress as an Internal State Variable. Acta Metall..

[21] Preston D.L., Tonks D.L., Wallace D.C. (2003). Model of Plastic Deformation for Extreme Loading Conditions. J. Appl. Phys..

[22] Ducobu F., Rivière-Lorphèvre E., Filippi E. (2017). On the Importance of the Choice of the Parameters of the Johnson–Cook Constitutive Model and Their Influence on the Results of a Ti6Al4V Orthogonal Cutting Model. Int. J. Mech. Sci..

[23] Storchak M., Rupp P., Möhring H.-C., Stehle T. (2019). Determination of Johnson–Cook Constitutive Parameters for Cutting Simulations. Metals.

[24] Johnson G.R., Cook W.H. (1985). Fracture Characteristics of Three Metals Subjected to Various Strains, Strain Rates, Temperatures and Pressures. Eng. Fract. Mech..

[25] Guo Y.B., Yen D.W. (2004). A FEM Study on Mechanisms of Discontinuous Chip Formation in Hard Machining. J. Mater. Process. Technol..

[26] Ling C., Ren X., Wang X., Li Y., Liu Z., Wang B., Zhao J. (2024). Dynamic Mechanical Properties and Modified Johnson–Cook Model Considering Recrystallisation Softening for Nickel-Based Powder Metallurgy Superalloys. Materials.

[27] Børvik T., Hopperstad O.S., Berstad T., Langseth M. (2001). A computational model of viscoplasticity and ductile damage for impact and penetration. Eur. J. Mech. A/Solids.

[28] Lu X., Lu Y., Wang F., Zhao C., Gao L., Si L. (2016). Research on surface residual stress of micro-milling nickel-based superalloy Inconel 718. Int. J. Nanomanuf..

